# Single‐cell dissection reveals immunosuppressive F13A1+ macrophage as a hallmark for multiple primary lung cancers

**DOI:** 10.1002/ctm2.70091

**Published:** 2024-11-27

**Authors:** Chenglin Yang, Jiahao Qu, Jingting Wu, Songhua Cai, Wenyi Liu, Youjun Deng, Yiran Meng, Liuqing Zheng, Lishen Zhang, Li Wang, Xiaotong Guo

**Affiliations:** ^1^ National Cancer Center/National Clinical Research Center for Cancer/Cancer Hospital & Shenzhen Hospital Chinese Academy of Medical Sciences and Peking Union Medical College Shenzhen China; ^2^ Southern University of Science and Technology Shenzhen City Guangdong Province China; ^3^ Department of R&D Hangzhou Repugene Technology Co., Ltd. Hangzhou China

**Keywords:** F13A1+ Mϕ, MPLC, scRNA‐seq, SPLC

## Abstract

**Background:**

The increasing prevalence of multiple primarylung cancers (MPLCs) presents challenges to current diagnostic and clinicalmanagement approaches. However, the molecular mechanisms driving MPLCdevelopment and distinguishing it from solitary primary lung cancers (SPLCs)remain largely unexplored.

**Methods:**

We performed a comparative single‐cell RNAsequencing (scRNA‐seq) analysis on tumour and adjacent para‐tumour tissues fromMPLC and SPLC patients to comparatively evaluate their immunological landscapes.Additionally, multiplex immunofluorescence (mIF) staining and independentvalidation datasets were used to confirm findings.

**Results:**

MPLCs and SPLCs share significant similarities in genetic, transcriptomic and immune profiles, suggesting common therapeutic strategies such as EGFR‐TKIs andICIs. Notably, an immunosuppressive macrophage subtype, F13A1+ Macrophage (Mϕ), is specifically enriched in MPLCs. This subtype overexpresses M2 macrophagemarkers and exhibits up‐regulation of SPP1‐CD44/CCL13‐ACKR1 interactions, indicatingits role in shaping the immunosuppressive tumour microenvironment and promotingtumour growth in MPLCs.

**Conclusions:**

This study unveils shared molecular mechanismsbetween MPLCs and SPLCs, while identifying MPLC‐specific cellular and molecularfeatures, such as the role of F13A1+ macrophages. The findings provide novelinsights into MPLC pathogenesis, supporting the development of targetedtherapeutic strategies.

**Key points:**

- Comparative scRNA‐seq analysis reveals significant similarities in genetic, transcriptomicand immune profiles between MPLCs and SPLCs.
- Identification of a unique immunosuppressive F13A1+ macrophage subtype, preferentially enriched in MPLCs, linked to immune evasion and tumourprogression.
- SPP1‐CD44/CCL13‐ACKR1 interactions are crucial in MPLC tumour microenvironment, indicating potential targets for therapeutic intervention.

## Graphical abstract

1. Comparative single‐cell RNA sequencing (scRNA‐seq) analysis reveals significant similarities in genetic, transcriptomic and immune profiles between multiple primary lung cancers (MPLCs) and solitary primary lung cancers (SPLCs).
2. Identification of a unique F13A1+ macrophage subtype, preferentially enriched in MPLCs, linked to immune evasion and tumour progression.
3. SPP1‐CD44/CCL13‐ACKR1 interactions are crucial in MPLC tumour microenvironment, indicating potential targets for therapeutic intervention.

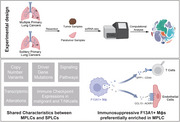

## 1 INTRODUCTION

Multiple primary malignant tumours (MPMT) refer to the simultaneous or sequential occurrence of more than one tumour lesions with different histological or morphological characteristics at different sites in the same individual.^1^ Lung cancer, among solid tumours, continues to be the leading cause of cancer‐related mortality worldwide, partially due to its high prevalence.^2^ Based on the number of lesions, primary lung cancer is classified into solitary primary lung cancer (SPLC) and multiple primary lung cancer (MPLC), with MPLC accounting for  .8%–14.5% of newly diagnosed lung cancers.^3^, ^4^, ^5^ Additionally, the annual likelihood of developing an additional primary lung cancer ranges from 1% to 3%, influenced by the time point of the initial treatment.^6^, ^7^, ^8^, ^9^ Epidemiological studies highlight several factors contributing to the increased susceptibility to multiple tumour lesions, including genetic predisposition, prior anti‐cancer treatment and environmental exposures such as smoking, alcohol and carcinogens.^10^ The rising incidence of MPLC in recent years can be attributed to advanced diagnostic techniques, increased life expectancy and improved surveillance of cancer patients.^11^

Despite remarkable advancements in the diagnosis, treatment and prognosis management of SPLC cases, MPLC presents several challenges in clinical practice. Firstly, accurately distinguishing MPLCs from intrapulmonary metastases or recurrent tumours is critical for determining appropriate treatment strategies. An accurate diagnosis often requires integrating several diagnostic modalities, including histopathological evaluation, radiological imaging and advanced methods such as genetic and molecular profiling.^12^, ^13^ Secondly, the genetic complexity and heterogeneity among MPLC patients necessitate individualised therapeutic approaches, as each lesion may exhibit unique molecular characteristics and treatment responses. Furthermore, there is currently no consensus or specific guidelines for the management of MPLC, highlighting the need for extensive research to explore its molecular mechanisms and to improve patient outcomes.

Recent work on MPLC has largely focused on their pathological and genomic alterations. Genomic profiling has uncovered considerable genetic heterogeneity among multiple lesions within the same patient, supporting the notion of independent origins.^14^, ^15^, ^16^ Critical cancer‐associated signalling pathways, including alterations in cell cycle regulation, immune‐related pathways and MAPK/ERK pathway, have also been implicated in the development and progression of MPLC.^17^ Of note, advances in next‐generation sequencing technologies have facilitated the identification of driver mutations and the administration of tyrosine kinase inhibitors (TKIs) in MPLC, which significantly improved clinical outcomes of patients.^15^ Despite these progress, effective treatment strategies for TKI‐resistant patients or for individuals without driver mutations remain poorly defined, underscoring the need for novel biomarkers to monitor the dynamic tumour microenvironment (TME) during tumour progression and to guide the development of effective treatments in clinical practice.

Cancer cells develop multiple mechanisms to circumvent anti‐tumour immune responses, one of which is through reprogramming the components within tumour immune microenvironment (TIME). In recent years, single‐cell RNA sequencing (scRNA‐seq) has become an invaluable tool for unravelling the intratumoural heterogeneity and dissecting common mechanisms in complex diseases like MPLC for its high resolution. Analysing scRNA‐seq data enables the identification of key molecular drivers shared among patients and allows the assessment of interactions between tumour cells, immune cells, stromal cells and other components within the TME. This is instrumental in uncovering potential targets for developing immunotherapy in MPLC. Recent scRNA‐seq studies have dissected the landscapes of tumour‐infiltrating immune cell, their developmental trajectories and regulatory networks in MPLC. One study reported two subtypes of malignant epithelial cells with distinct functions in MPLC: State 1 is involved in immune response, and State 2 is associated with cell death.^18^ Another single‐cell transcriptomic analysis has highlighted increased heterogeneity among nodules in lung adenocarcinoma progression, emphasising the significant roles of CD8+ cytotoxic T cells, NK cells, as well as myeloid cells.^19^ Although genomic, DNA methylation and bulk transcriptomic studies have indicated differential characteristics between SPLC and MPLC,^20^ a comprehensive analysis that simultaneously characterises the transcriptomic landscapes of SPLC and MPLC at the single‐cell resolution remain notably limited.

In our study, we aim to utilise scRNA‐seq analysis to explore the cellular heterogeneity, genomic landscape and immune characteristics of MPLCs and perform a direct comparison to SPLCs. By systematically evaluating the transcriptional profiles of all cell populations in MPLCs and SPLCs, we seek to identify unique molecular signatures associated with MPLCs and shed light on the factors driving their development and progression. Ultimately, our objective is to advance our knowledge of these complex tumours, inform clinical decision‐making and facilitate the development of personalised treatment strategies for MPLC patients.

## 2 METHODS

### 2.1 Sample acquisition

The Medical Research Ethics Committee of National Cancer Center, the National Clinical Research Center for Cancer and the Cancer Hospital & Shenzhen Hospital, all affiliated with the Chinese Academy of Medical Sciences and Peking Union Medical College. Written informed consent was obtained from all subjects. The ethical approval number was KYKT2023‐32‐1. Newly diagnosed, eight untreated patients with respiratory malignancies, including five MPLCs and three SPLCs, were prospectively recruited once referred. Freshly resected tumour tissues and matched normal tissues were obtained from lung cancer patients following surgical resection. Normal lung tissue was also evaluated, and only lung tissue without cancer cell infiltration was included in the scRNA‐seq analysis. The clinical information of all patients is shown in Table S1.

### 2.2 Tissue processing and single‐cell RNA sequencing

Samples of freshly obtained tissues were placed into GEXSCOPETM tissue Preservation Solution and maintained at 4°C. All specimens were then cleansed with Hanks Balanced Salt Solution for three times and cut into small pieces, which were followed by digesting tissue fragments in Tissue Dissociation Solution at 37°C for 15 min with gentle shaking. The resulting cell suspension was passed through a 40‐µm sterile strainer to remove debris. Following this, the cell suspension was centrifuged at 1000 rpm, 4°C for 5 min, and the resulting cell pellets were resuspended in the phosphate buffered saline (PBS). To eliminate red blood cells, 2 mL of Red Blood Cell Lysis Buffer was added and was incubated at 25°C for 10 min, and centrifuged at 1000 rpm for 5 min. The cell pellet was reconstituted, and the cell concentration was measured by the TC20 automated cell counter. The suspension was then prepared at a concentration of 1 × 10^5^ cells/mL and subsequently introduced into a microfluidic platform. For the construction of scRNA‐seq libraries, the Single Cell RNA‐seq Library Kit was utilised following the manufacturer's protocol. Sequencing was performed on an Illumina HiSeq X Ten instrument.

### 2.3 Data preprocessing and scRNA‐seq quantifications

Quality control measures were applied to the raw sequencing data, including quality evaluation, trimming, alignment and transcript counting, based on *FastQC* (version 0.11.7), *STAR* (version 2.5.3a)^21^ and *featureCounts* (version 1.6.2)^22^ respectively. The clean reads were aligned to the reference genome GRCh38 from the Ensembl database. The aligned reads were further processed to obtain gene expression counts, and the resulting count matrices were used for downstream analysis. The raw unique molecular identifier (UMI) count matrices were converted into a Seurat object using the R package *Seurat* (version 3.2.3),^23^ followed by filtering steps to: (1) remove cells with a fewer than 200 unique detected genes or more than 5000; (2) discard cells exceeding 30 000 UMIs; (3)remove cells having over 30% mitochondrial content; (4) regress out cell cycle genes. After filtering, 327 611 cells remained for downstream processing.

### 2.4 Dimensionality reduction and clustering analysis

Initially, we normalised the integrated data through the *NormaliseData()*, then identified the top 2000 highly variable genes with the *FindVariableFeatures()*. Simultaneously, all genes were scaled with the *ScaleData* function. To reduce dimensionality, the *RunPCA* was applied for principal component analysis (PCA). We set the dimensional parameter to 30 and used the *FindNeighbours* and *FindClusters* functions to identify clusters. Subsequently, the first 30 principal components were selected to reduce dimensionality using Uniform Manifold Approximation and Projection (UMAP) and t‐distributed stochastic neighbour embedding (t‐SNE) method. Next, batch effects were corrected at the sample level using the *runHarmony* function from the Harmony package (version 0.1.0)^24^ with the parameter *group.by.vars = ‘Sample.ID’* to address variability across samples arising from processing and sequencing. For sub‐clustering, we followed a similar approach that involved identifying variable genes, reducing dimensionality and further cluster cells within the restricted subset.

### 2.5 Cell type identification

To label cell clusters, we employed the *FindAllMarkers()* function, which utilises a *Wilcoxon rank‐sum test* adjusted by *Bonferroni correction* to identify differentially expressed genes (DEGs) with strong discriminatory abilities between the groups. The annotation of cell groups was then accomplished by leveraging the information from the DEGs and well‐established markers reported in the literature. Epithelial cells were marked by high expression levels of *EPCAM, KRT8* and *CAPS*, and the remaining cell types were annotated with the markers: T/NK cells (*CD3D, CD3E*, *CD3G* and *NKG7*), plasma (*JCHAIN, IGKC* and *IGHG1*), B cells (*CD79A, MS4A1* and *CD19*), myeloid cells (*LYZ, CD14* and *SPP*1), cancer‐associated fibroblasts (CAFs; *DCN, LUM* and *COL1A1*), mast cells (*KIT, CPA3* and *MS4A2*) and endothelial cells (*VMF, PECAM1* and *CALCRL*).

### 2.6 Analysis of gene expression differences and associated pathways

To identify DEGs between two sets of clusters, we employed the *wilcoxauc* function from the *presto* package. This method facilitated the identification of DEGs by conducting the Wilcoxon rank‐sum test and the area under the receiver operating curve (AUC).

The AUC statistic was used to rank the genes and served as input for the subsequent Gene Set Enrichment Analysis (GSEA).^25^ GSEA was applied to evaluate the enrichment of predefined gene sets associated with specific biological processes. For each gene set, the enrichment score (ES) was access on a pre‐ranked gene list derived from the two samples or clusters. The calculated ES values were then normalised to yield the normalised enrichment score (NES). Furthermore, the false discovery rate (FDR) was determined by comparing the observed distribution of NES to the null distribution, taking into consideration the size of the gene set.

Gene ontology (GO) enrichment analysis was performed using Enrichr (https://maayanlab.cloud/Enrichr/) online tool with input species set to *Homo sapiens* and using standard parameters.^26^ And the top ten terms were retained according to the adjusted *p* value.

### 2.7 Group distribution of clusters

To analyse the distribution of sub‐clusters, odds ratios (ORs) were computed to indicate preferences.^27^ Given a contingency table of sub‐clusters by groups, we first apply Fisher's exact test to obtained OR and corresponding *p* value. Specifically, a 2 by 2 contingency table was constructed for each combination of meta‐cluster *i* and group *j*. Fisher's exact test was then performed on the contingency table to obtain the OR and *p* value. The *p* values were corrected using the *Benjamini–Hochberg (BH)* method implemented in the R function *p.adjust*. Adjusted *p* values below  .01 were deemed significant for ORs greater than 1.5 or less than  .5. Therefore, one cluster was identified as being enriched in a specific group if OR > 1.5, while a lower OR (<.5) indicated a strong inclination for cluster *i* to refrain from distributing in group *j*.

### 2.8 Copy number variation and clonal evolution analysis

For each tumour lesion, the epithelial cells were considered as the putative tumour epithelium dataset, and 3000 T/NK cells from paracancerous tissues was utilised as reference. *InferCNV*, which employs a Hidden Markov Model (HMM), was employed to predict the level of copy number variations (CNVs). To correct the results, a Bayesian Network Model was implemented to determine the likelihood of alteration status in each cell and the entire CNV region. To quantitatively evaluate the CNV level of each single cell, a CNV score was defined as the quadratic sum of CNV values across the genome. Cells with CNV scores exceeding two standard deviations above the reference's mean were identified as malignant cells. In order to simplify the analysis based on location, the cytoband information from GRCh38 was utilised to map each CNV to its corresponding p‐ or q‐arm alteration. Each CNV was further labelled as a gain or a loss. Subsequently, subclones containing identical arm‐level CNVs were collapsed. To generate evolutionary trees, UPhyloplot2 (version 2.3)^28^ was employed with default parameters.

### 2.9 Module score calculation

To assess the activity of specific gene sets within the single‐cell populations, the *AddModuleScore()* function was applied. This function calculates a module score for each cell based on the mean expression of a set of genes, relative to a control gene set.

### 2.10 Cell–cell interaction network

To explore the communications among various subtypes, we employed CellChat (version 1.1.3) for cell–cell interaction analysis, which utilises a robust statistical framework combined with a curated database of ligand–receptor pairs.^29^ To ensure the reliability of the identified interactions, we applied stringent criteria for their retention. Only interactions that met the following conditions were retained: (1) the ligand and receptor genes had to be expressed in over 10% of both signalling and target cells, and (2) the ligand's gene expression had to meet a significant log2 fold change (log2FC), with an interaction threshold set at >.1 and a statistically significant adjusted *p* value of <.05.

### 2.11 Gene co‐expression correlations

Gene functionality was predicted by co‐expression analysis between candidate genes and functionally known genes within specific biological contexts based on Spearman's correlation coefficient.

### 2.12 Statistical analysis

All statistical analyses were performed using R software. The Wilcoxon rank‐sum test was employed to compare two groups of samples. Statistical significance was indicated: ^*^ *p* < .05, ^**^ *p* < .01, ^***^ *p* < .001.

### 2.13 Pseudotime reconstruction and trajectory inference

To study dynamic biological process, including the transitions and evolutionary trajectory of monocyte/macrophage and dendritic cells (DCs), Monocle (Version 2.28.0) was employed.^30^, ^31^, ^32^ The *NewCellDataSet* function was employed to generate an object for monocle from transcript count data of the selected cell populations. Normalisation of mRNA recovery differences and setup for differential expression analysis were achieved with the *estimateSizeFactors* and *estimateDispersions* functions. Genes expressed in at least 10% of cells and with *p* < .01, as determined by *differentialGeneTest* function, were selected for further analysis due to their significant variation in expression along the trajectory. To facilitate easier visualisation and interpretation, the *reduceDimension* function was employed to reduce the data to two dimensions. Subsequently, the *orderCells* function was utilised to order the cells along the trajectory, using the *root_state* argument to specify the starting point based on the expression of early cell type‐specific genes. Once the cells were ordered, the trajectory was visualised in the reduced dimensional space using the function *plot_cell_trajectory*, with *colour_by = ‘Cell types’*, *‘Pseudotime’* or *‘State’*. To identify major expression patterns along the pseudotime trajectory, the top 1000 pseudotime‐related genes was selected using the *differentialGeneTest* function, with the parameter *‘fullModelFormulaStr’ to ‘∼sm.ns(Pseudotime)’*, and these genes were then subsequently grouped into six clusters using k‐means clustering. Finally, the genes were visualised using the *plot_pseudotime_heatmap* and *plot_genes_branched_heatmap* functions with default parameters.

To further validate the trajectory inferred from Monocle2, we applied the Slingshot algorithm^33^ as an independent method for pseudotime and trajectory inference. Using the same reduced dimensional data obtained from the Monocle2 analysis, we applied the *slingshot()* function to infer pseudotime trajectories based on predefined cell types, and the starting cluster was manually defined to match the initial state identified in the Monocle2 analysis. The *slingshot()* function was employed with default settings, and the inferred trajectories were visualised using the *plot()* and *lines()* functions to compare pseudotime progression across lineages. Pseudotime ordering of cells was used to validate whether the differentiation patterns were consistent between Slingshot and Monocle2 methods.

### 2.14 Survival analysis

Microarray data GSE31210^34^, ^35^ for lung cancer expression and survival information were downloaded from the Gene Expression Omnibus (GEO), which includes 226 lung cancer samples, for subsequent survival analysis. DEGs between the cluster of interest and other clusters were identified and ranked by log2FC. The top 10 genes were selected as marker genes for each cluster. For each bulk RNA‐seq sample in GSE31210, the expression values of these marker genes were log2‐transformed and then standardised using *z*‐score. The mean of the standardised expression values of these 10 marker genes was calculated for each sample. Based on the median of this mean expression, samples were divided into high and low expression groups. Kaplan–Meier analysis and log‐rank tests were performed using the *survminer* and *survival* R package to evaluate disease‐free survival (DFS) differences between groups. Cox proportional hazards models were employed to estimate hazard ratios (HRs) with 95% confidence intervals and assess prognostic significance, with significance evaluated by the log‐rank test.

Next, CIBERSORTx (https://cibersortx.stanford.edu/)^36^ was used to estimate cell‐type proportions in bulk RNA‐seq samples via deconvolution, using single‐cell RNA‐seq data as the reference. A signature matrix was created by averaging gene expression profiles of annotated cell clusters. This matrix was applied in ‘absolute mode’ with batch correction to calculate cell‐type fractions across bulk samples. Then the samples were stratified into high and low groups based on the median fraction of each cell type. Kaplan–Meier analysis, log‐rank tests and Cox proportional hazards models were used to evaluate DFS differences and the prognostic impact of these cell types.

### 2.15 Multiplex immunofluorescence staining

Tissue sections from MPLC and SPLC specimens, preserved in formalin and embedded in paraffin (FFPE) were deparaffinised and rehydrated using a series of graded alcohol. Heat‐induced antigen retrieval was performed using citrate buffer (pH 6.0) for optimal antigen exposure. To prevent non‐specific binding, a blocking solution was applied for 1 h at room temperature. A three‐colour fluorescence kit based on tyramide signal amplification (TSA) was utilised according to the manufacturer's protocol (Akoya Biosciences). Briefly, FFPE tissue sections were first treated with primary antibodies, followed by corresponding secondary antibodies and TSA solution. The primary antibodies used in conjunction with Opal dyes were as follows: anti‐CD68 (CST, catalogue number: 97778) with Opal 570 (yellow), anti‐F13A1 (Abcam, catalogue number: ab76105) with Opal 620 (magenta), and anti‐pan cytokeratin antibody (Abcam, catalogue number: ab7753) with Opal 520 (green). Multiplex staining involved iterative cycles comprising antigen retrieval, protein blocking, antigen labelling and signal amplification steps. Following the final TSA cycle, a 1:1000 dilution of 4′,6‐diamidino‐2‐phenylindole (DAPI) was counterstained for 5 min at room temperature. Subsequently, coverslips were mounted onto the stained sections using an anti‐fade mounting medium to preserve fluorescence signals. Fluorescent images were captured with an Olympus CX23 microscope and visualised with CaseViewer imaging analysis software v.2.4. Quantification analysis on the entire tissue sections was conducted using Halo HighPlex FL v.4.0.3 module (Indica Labs), focusing on comparing the F13A1^+^CD68^+^ cell fraction between MPLCs and SPLCs.

## 3 RESULTS

### 3.1 Overall characteristics of the cellular composition in MPLCs and SPLCs

**FIGURE 1:**
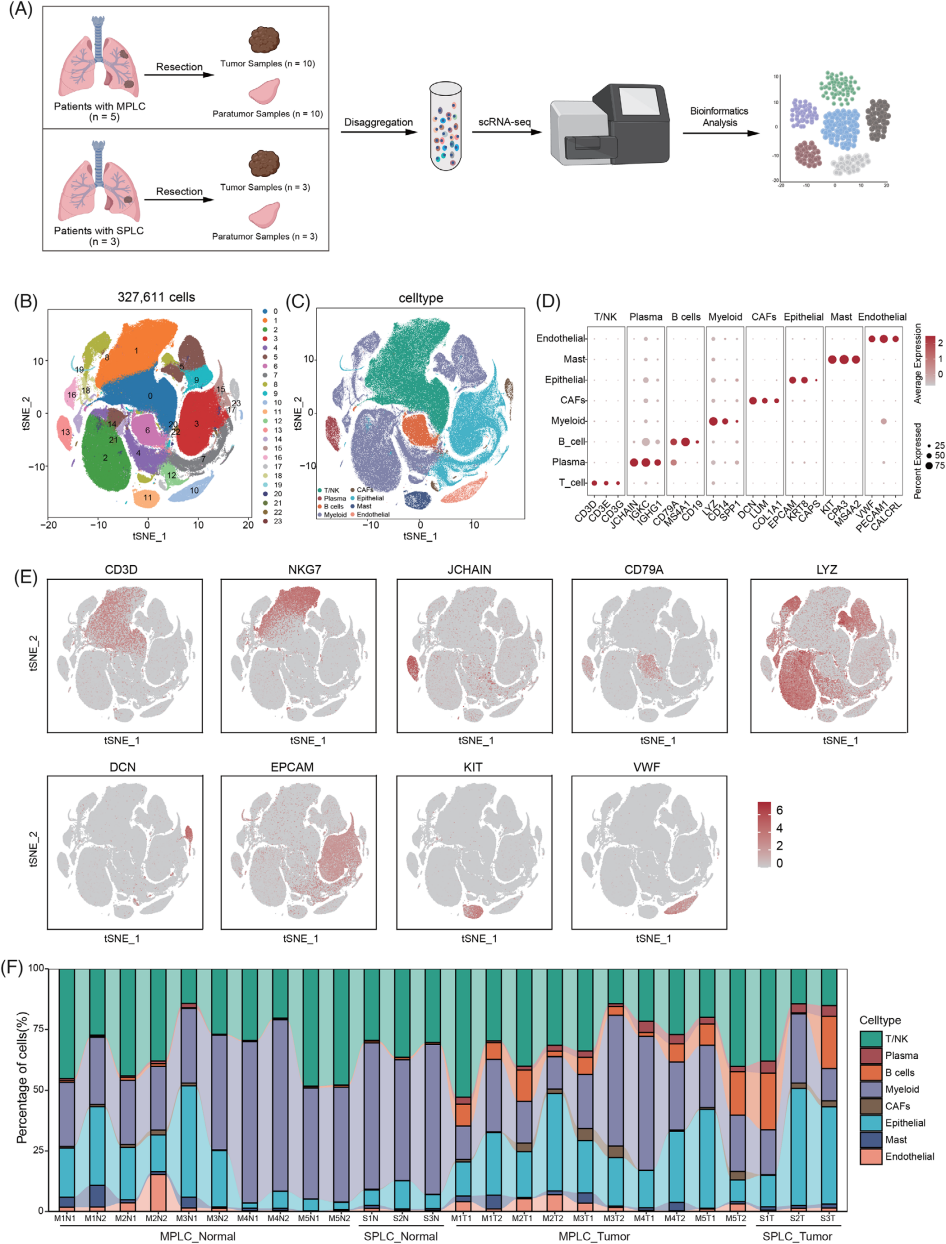
Overall characteristics of the cell cluster composition in multiple primary lung cancer (MPLC) versus solitary primary lung cancer (SPLC). (A) Schematic representation of the experimental workflow. Tumour and normal samples were resected from patients with MPLC and SPLC. Tumour samples were collected from 10 MPLC sites (MT) and three SPLC sites (ST), along with corresponding normal samples from MPLCs (MN) and SPLCs (SN). The samples were then disaggregated and subjected to single‐cell RNA sequencing (scRNA‐seq) followed by bioinformatics analysis to identify and characterise different cell types. (B) t‐Distributed stochastic neighbour embedding (t‐SNE) projection of 327 611 cells based on their gene expression profiles by their cluster assignment. Each colour represents a different cell cluster. (C) t‐SNE projection of all the cells coloured and labelled by inferred cell types. (D) Dot plots showing the average expression and per cent expression of canonical markers across different cell types. Marker genes include *CD3D* (T cells), *NKG7* (NK cells), *JCHAIN* (plasma cells), *CD79A* (B cells), *LYZ* (myeloid cells), *DCN* (CAFs), *EPCAM* (epithelial cells), *KIT* (mast cells) and *VWF* (endothelial cells). (E) Feature plots showing the scaled expression of selected marker genes (*CD3D*, *NKG7*, *JCHAIN*, *CD79A*, *LYZ*, *DCN*, *EPCAM*, *KIT*, *VWF*) across all cells. Red indicates higher expression levels. (F) Stacked bar plot showing the percentage of different cell types in each sample group. The groups include MPLC normal (MN1–MN5), SPLC normal (SN1–SN3), MPLC tumour (MT1–MT7) and SPLC tumour (ST1–ST3).

To thoroughly understand the TME, and to investigate the heterogeneity and commonness among tumour lesions from MPLCs and SPLCs, we conducted a comprehensive analysis of 10 tumour samples from five MPLC patients, with each patient presenting two separate lung tumour lesions, and three tumour samples from three SPLC patients. For comparative purposes, we also collected adjacent normal tissue samples corresponding to each tumour lesion (Figure 1A and Table S1). Used the established cell markers outlined in Section 2, we classified 327 611 cells into T/NK cells (99 152 cells, 30.27%), plasma cells (5963 cells, 1.82%), B cells (18 350, 5.60%), myeloid cells (118 556, 36.19%), CAFs (3908, 1.19%), epithelial cells (68 911, 21.03%), mast cells (6272, 1.19%) and endothelial cells (6499, 1.98%) (Figures 1B–E, S1D and Table S2).

Compared to adjacent normal tissues, tumour tissues from both MPLCs and SPLCs exhibited higher proportions of certain cell populations. Specifically, the presence of plasma (.49% and  .13% in normal tissues from MPLC and SPLC, respectively; 2.57% and 4.37% in tumour tissues), B cells (.62%,  .78% in normal vs. 7.66%, 15.77% in tumour), CAFs (.56%,  .16% in normal vs. 1.91%, 1.74% in tumour) and epithelial cells (16.08%, 7.74% in normal vs. 24.17%, 34.86% in tumour) were more pronounced in tumour tissues (Figures 1F and S1B,C). Conversely, myeloid cells were more enriched in adjacent normal tissues compared to tumour tissues. These cells are integral to the innate immune system and play critical roles in inflammation and immune surveillance. The lower proportion of myeloid cells in tumour tissues suggested an altered immune landscape within the TME.

The cellular composition of multiple primary tumours and single‐primary tumours showed significant differences compared to adjacent normal tissues, notably with increased proportions of plasma cells and B cell, both of which are key components of the immune system. The enrichment of CAFs and epithelial cells in tumour tissues reflects the tumour‐promoting processes and the expansion of the tumour epithelium, which is responsible for the uncontrolled growth and invasion characteristic of cancer. The cell type composition similarities between MPLCs and SPLCs highlighted common characteristics and underlying mechanisms in tumour development and progression.

### 3.2 Common genetic drivers in MPLCs and SPLCs

Although the epithelial population mostly consists of tumour cells, it may include residual normal cells. To discern malignant cells from normal cells within the epithelial cell population, we employed the *inferCNV* algorithm, which leverages the presence of large‐scale somatic CNV events, to identify malignant cells. Tumour‐derived epithelial cells and 3000 T/NK cells from adjacent normal tissues were used as inputs and reference controls, respectively (Figure S2A).

Out of 47 041 epithelial cells from tumour tissues, 40 291 cells were classified as malignant based on their high CNV scores (Figure S2B). We identified specific gene expression pattern for malignant cells in MPLCs or SPLCs compared to their corresponding non‐malignant epithelial cells (Figure S2C). The analysis revealed that malignant cells both in MPLCs and SPLCs down‐regulated expression of several tumour suppressor genes associated with non–small‐cell lung cancer (NSCLC; e.g., *WIF1*, *TSC22D1*, *SMARCA5*, *SFTPC*, *SFTPA2*, *SFTPA1*, *PTPN13*, *MFSD2A*, *HHIP*, *DUOX1*, *ABCA3* and *CACNA2D2*),^37^, ^38^, ^39^, ^40^, ^41^, ^42^, ^43^, ^44^, ^45^, ^46^ while showing increased expression of LUAD markers and epithelial cell growth factors (e.g., *KRT7*, *KRT18*, *KRT8* and *GDF15*), as well as poor prognostic and immunological biomarker of LUAD (e.g., *PPP1R14B*, *NQO1*, *SNRPE*, *TACSTD2*, *SERINC2* and *GOLM1* (Tables S3 and S4).^47^, ^48^, ^49^, ^50^, ^51^ Consistent with these observations, GSEA analysis showed that hallmarks associated with tumourigenesis, including epithelial–mesenchymal transition and P53 Pathway, were significantly enriched in both MPLCs and SPLCs, suggesting common underlying mechanisms in their tumour development and progression (Figure S2D and Table S5). Furthermore, immune checkpoint genes (ICGs), such as *LGALS3*, *HLA‐E* and *HLA‐C*, were robustly expressed in both MPLCs and SPLCs, suggesting the similar immune evasion mechanisms. This indicates that specific immune checkpoint inhibitors (ICIs) could potentially exert their therapeutic effects in both cancers (Figure S2E).

Based on the compiled CNV results, we employed the UPhyloplot2 plotting algorithm to generate clonality trees for each tumour lesion, allowing us to visualise their evolutionary relationships and clonal structures. Remarkably, consistent CNV events were observed in both MPLCs and SPLCs, such as gain of the long arm of chromosome (5q), gain of chromosome 7, loss of chromosome 17, loss of chromosome 2, loss of chromosome 3, gain of chromosome 8 and so forth (Figure S2F,G). Analysis of predicted CNV genes using the HMM revealed consistent alterations in driver genes across multiple samples. These alterations include amplification of *SQSTM1*, *EGFR* and *MYC*, deletion of *MET*, *TP53*, *PHF23*, *ERBB2*, *VHL* and *RB1*, indicating the presence of shared driver genes (Figure S2G). These shared alterations are known to be associated with crucial signalling pathways that contribute to tumour growth and progression. Thus, targeting clone populations harbouring these alterations could serve as an effective therapeutic strategy for these tumours, irrespective of whether there are multiple or single‐primary tumours.

These findings suggest a shared clonal origin between MPLCs and SPLCs, and underscore the potential convergence in the underlying pathways of tumour development. The presence of common genetic disruptions on key oncogenes or tumour suppressor genes suggests that targeted interventions focusing on these specific genetic alterations could potentially yield effective in both single‐primary and multi‐primary tumours. Despite their shared clonal origins, each tumour lesion exhibits distinct genetic and molecular features. Further research is warranted to investigate whether these features are associated with treatment efficacies and clinical outcomes.

### 3.3 Shared characteristics in tumour‐infiltrating T/NK cells across MPLCs and SPLCs

**FIGURE 2:**
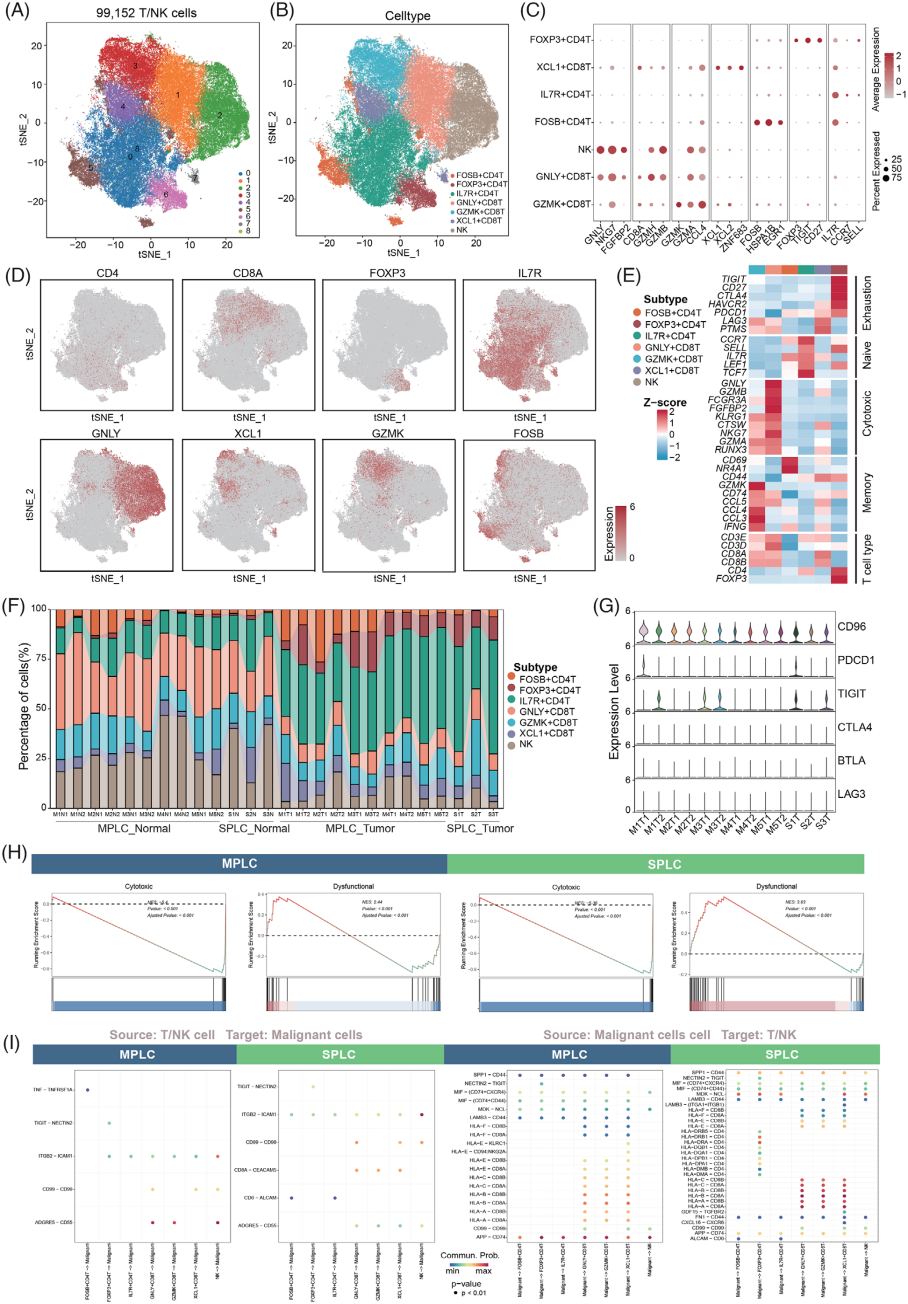
Characterisation of tumour‐infiltrating T/NK cells. (A) t‐Distributed stochastic neighbour embedding (t‐SNE) plot of 90 180 T/NK cells by their cluster assignment. (B) t‐SNE projection of all the T/NK cells coloured and labelled by inferred cell types. (C) Dot plot showing the average expression and per cent expression selected cell type‐specific markers across different T/NK cell subtypes. The size of dots represents the fraction of cells expressing a particular marker, and the intensity of the colour indicates the levels of average mean expression. (D) Feature plots showing the scaled expression of canonical marker genes, which were denoted in C. (E) Heatmap depicting the expression levels of exhaustion, naive, cytotoxic and memory markers across different T/NK cell subtypes. *Z*‐scores are used to indicate the relative expression levels. (F) Stacked bar plot showing the percentage of different T/NK cell subtypes in each sample group. (G) Stacked violin plots illustrating the expression levels of immune checkpoint molecules (*CD96*, *PDCD1*, *TIGIT*, *CTLA4*, *BTLA*, *LAG3*) in T/NK cell subtypes across different sample groups. (H) Gene Set Enrichment Analysis (GSEA) for T/NK cells. The left plot compares T/NK cells from multiple primary lung cancer (MPLC) tumours to those from MPLC normal tissues, and the right plot compares T/NK cells from solitary primary lung cancer (SPLC) tumours to those from SPLC normal tissues. (I) Interaction plots depicting the inferred cell–cell communication between T/NK cells and malignant cells. The left plot shows interactions with T/NK cells as the source and malignant cells as the target, while the right plot shows interactions with malignant cells as the source and T/NK cells as the target, for both MPLC and SPLC samples. The dot size represents *p* value, and the colour indicates the probability of ligand–receptor interaction.

Following quality control filtering, a total of 99 152 T and NK cells were obtained from tumours and adjacent normal tissues. Graph‐based clustering analysis revealed the presence of several distinct T and NK cell clusters, each displaying unique marker gene expression profiles associated with specific T/NK cell subsets, including three cluster of CD4+ T cell, three clusters of CD8+ T cells and one NK cluster (Figure 2A,B). Cluster 2 was identified as NK cells, characterised by elevated expression levels of NK cell signatures such as *NKG7, GNLY, FCGR3A, FGFBP2* and *KLRD1*. Among the CD4+ T cell clusters, IL7R+ CD4+ T cells exhibited gene expression patterns associated with naive T cells, including *CCR7, IL7R, LEF1* and *SELL*. FOXP3+ CD4+ T cells were defined by elevated levels of canonical Treg markers such as *FOXP3, TIGHT* and *CD27*. Cluster 6 was notable for its expression of stress response genes, including *FOSB* and several heat shock proteins (HSPs) such as *HSPA1A, HSPH1, HSPA1B* and *HSPA6*. Within the CD8+ T cell clusters, GNLY+ CD8+ T cells displayed a gene expression profile characteristic of effector CD8+ T cells (CD8+ Teff), with high levels of cytotoxic genes like *GZMB*, *GNLY*, *FCGR3A*, *FGFBP2*, *KLRG1*, *CTSW*, *NKG7*, *RUNX3* and *GZMA*. GZMK+ CD8+ T cells expressed high levels of both cytotoxic and memory‐related transcripts, including *GZMA, KLRG1* and chemokines such as *CCL3*, *CCL4* and *CCL5*, indicative of effector memory T cells (Tem). XCL1+ CD8+ T cells exhibited an activation signature characterised by the expression of *PDCD1*, *HAVCR2*, *LAG3* and *PTMS*, annotated as exhausted CD8+ T cells (CD8+ Tex; Figures 2C–E and S3D).

A similar distribution of typical T/NK cell types was observed in tumour tissues derived from patients with MPLC and SPLC (Figures 2F and S3B). In comparison to normal tissues, there was a notable enrichment of naïve CD4+ T cells and regulatory T cells (regs) within the tumour tissues, irrespective of their MPLC or SPLC origin, while NK and CD8+ Teff cells were mainly distributed in adjacent normal tissue. At molecular level, certain ICGs and T cell exhaustion‐related markers, such as *CD96* and *TIGIT*, were expressed in both multiple primary and single‐primary patients (Figure 2G). To investigate the functional characteristics of T cells in MPLCs and SPLCs, GSEA analysis was applied. In T/NK cells from both MPLCs and SPLCs, genes associated with the T cell dysfunction pathway were highly enriched compared to T/NK cells from normal tissues, while the T cell cytotoxic pathway was largely suppressed (Figure 2H and Table S5). Further analysis revealed that exhaustion scores in CD8+ Tex cells and proliferation scores in T cells were comparable between MPLC and SPLC groups, with no significant difference in their median scores (Figure S3E,G). In contrast, cytotoxic scores for CD8+ Teff cells were higher in MPLC compared to SPLC, indicating a potentially more active cytotoxic potential in MPLC (Figure S3F). These findings align with previous studies showing that tumour‐infiltrating T cells primarily acquire an exhausted phenotype within the TME.^52^, ^53^, ^54^ These shared phenotypic and functional features of T cell infiltration suggest a common mechanism underlying decreased effector cytokines production and cytotoxicity, elevated and persistent expression of multiple inhibitory receptors in both types of tumours. Reinvigorating T cell function could profoundly impact the efficacy of immunotherapy in patients with both multiple and solitary primary tumours.

To further understand how T/NK cells directly impact on tumourigenesis, we applied *CellChat* to investigate the interplay between T/NK subsets and malignant cells in MPLCs and SPLCs (Figure 2I). Our findings revealed that as signal senders, T cells from both tumour types exhibited highly similar interaction patterns with malignant cells. Specifically, common ligand–receptor pairs, such as the *ITGB2‐ICAM1* pair, were universally involved in the interactions between most types of T/NK cells and malignant cells.^55^, ^56^ Ligand–receptor pairs including *CD99‐CD99* and *ADGRE5‐CD55* were predominantly identified in interactions between CD8+ T cells and NK cells with malignant cells, while Tregs mainly crosstalk with tumour cells via the *TIGIT–NECTIN2* interaction. *NECTIN2* (*CD122*) is overexpressed in various cancers,^57^, ^58^, ^59^ and *TIGIT* is commonly expressed in most Tregs in humans as a co‐inhibitory molecule.^60^, ^61^, ^62^, ^63^ The presence of the *TIGIT–NECTIN2* interaction in both tumour types indicates that the activation of immunosuppressive ligand–receptor pairs in the TME is irrespective of the number of primary tumours. As signal receivers, T cells also demonstrated analogous interaction patterns with malignant cells in both tumours. Specifically, the involvement of human leukocyte antigen (HLA) class I molecules (such as *HLA‐A*, *HLA‐B*, *HLA‐C*, *HLA‐E* and *HLA‐*F) and *CD8A/B* was consistently observed in the interactions between malignant cells and CD8 T cells in both tumour types. Furthermore, ligand–receptor pairs including SPP1‐CD44, *MIF‐(CD74+CD44)*, *MIF‐(CD74+CXCR4)*, *MDK‐NCL*, *LAMB3‐CD44* and *APP‐CD74* were identified in the interactions between nearly all subsets of T/NK cells and malignant cells. It is worth noting that the interaction between HLA‐II molecules (such as *HLA‐DRA*, *HLA‐DRB1*, *HLA‐DRB5*, *HLA‐DQB1*, *HLA‐DPB1*, *HLA‐DPA1*, *HLA‐DMB* and *HLA‐DMA*) and the receptor *CD4* was exclusively detected in the cell communication between malignant cells from single‐primary tumours and Tregs.

Overall, our study uncovered a number of shared characteristics in tumour‐infiltrating T/NK cells across both MPLCs and SPLCs, along with nuanced differences. These characteristics encompassed a comprehensive analysis of cell type distribution, phenotypic, transcriptional and functional attributes and the intricate interplay between T/NK cells and malignant cells within the TME.

### 3.4 F13A1+ Mϕs and CD1C+ cDC2 are MPLC‐specific myeloid subsets

**FIGURE 3:**
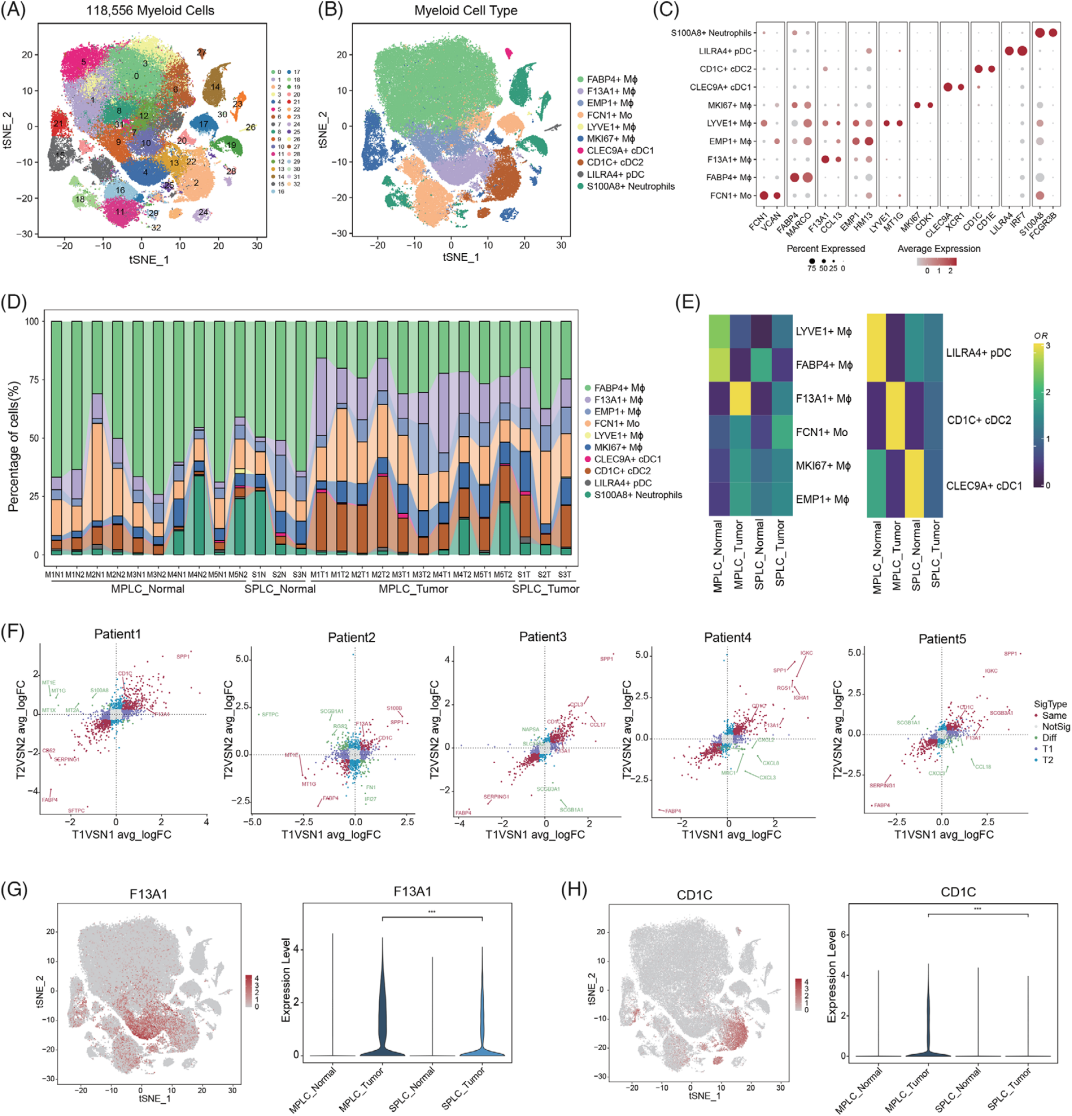
Myeloid landscape in multiple primary lung cancer (MPLC) versus solitary primary lung cancer (SPLC). (A) t‐Distributed stochastic neighbour embedding (t‐SNE) plot showing the clustering of 118 556 myeloid cells based on their gene expression profiles. Each colour represents a different cluster. (B) t‐SNE projection of all the myeloid cells coloured and labelled by inferred cell types. The major cell types include FABP4+ Mϕ, F13A1+ Mϕ, EMP1+ Mϕ, LYVE1+ Mϕ, FCN1+ Mo, MKI67+ Mϕ, CLEC9A+ cDC1, CD1C+ cDC2, LILRA4+ pDC and S100A9+ neutrophils. (C) Dot plot showing selected cell type‐specific markers across all myeloid cell types. The size of each dot represents the fraction of cells expressing a particular marker, and the colour intensity indicates the average expression level. (D) Stacked bar plot showing the proportion of 10 myeloid cell types across 26 samples. The groups include MPLC normal (MN1–MN5), SPLC normal (SN1–SN3), MPLC tumour (MT1–MT7) and SPLC tumour (ST1–ST3). (E) Heatmap showing the odds ratios (ORs) of Mo/Mϕ meta‐clusters (left) and dendritic cell (DC) meta‐clusters (right) of occurring in each group. An OR > 1.5 indicates a preference for the meta‐cluster to distribute in the corresponding group. (F) Scatter plots showing the gene expression differences between two distinct tumour lesions and their corresponding adjacent normal tissue in the cohort of five MPLC patients. The *x*‐axis represents the average log2 fold change (avg_logFC) calculated by comparing the first tumour lesion and normal tissue (T1 vs. N1) within each patient. Similarly, the *y*‐axis depicts the avg_logFC comparing the second tumour lesion and normal tissue in the other affected lobe (T2 vs. N2) of each patient. Dot colour indicates the significance type. (G) t‐SNE plots (left) showing the expression levels of *F13A1* in myeloid cell types. Violin plot (right) shows differential expression of *F13A1* across four groups, with a Wilcoxon test comparing tumour tissues from MPLCs and SPLCs. ^***^ *p* < .001. (H) t‐SNE plots (left) showing the expression levels of *CD1C* in myeloid cell types. Violin plot (right) shows differential expression of *CD1C* across four groups, with a Wilcoxon test comparing tumour tissues from MPLCs and SPLCs.

Tumour‐infiltrating myeloid cells are essential components of the TME. In our study, we performed a comprehensive analysis of 118 556 myeloid cells, identifying 33 distinct clusters (Figure 3A). To annotate individual immune cell sub‐populations, we used ‘*FindAllMarkers*’ in Seurat function for differential gene expression analysis. Feature genes for sub‐cluster characterisation were selected based on following criteria: expression in at least 25% of the population, a significant difference (adjusted *p* value <.05) calculated by the Wilcoxon test, and a log2FC >.25. In doing so, myeloid cells were categorised into nine clusters: six monocyte/macrophage (Mo/Mϕ) subtypes (FABP4+ Mϕ, F13A1+ Mϕ, EMP1+ Mϕ, FCN1+Mo, LYVE1+Mϕ and MKI67+Mϕ; Table S6), three DC subtypes (CLEC9A+ conventional type 1 DCs [cDC1], CD1C+ conventional type 2 DCs [cDC2] and LILRA4+ plasmacytoid DCs [pDCs]; Figure 3B,C and Table S7), and S100A8+ neutrophils.

To assess the distribution patterns of these myeloid subsets, we conducted OR analysis on the aforementioned Mo/Mϕs and DCs. The analysis revealed a strong distribution preference of F13A1+ Mϕ and CD1C+ cDC2 in tumours from MPLCs, as compared to tumours from SPLCs and normal tissues from both MPLCs and SPLCs (Figure 3D,E). This finding implies that these specific myeloid subtypes may contribute to the distinct characteristics and behaviour of multiple primary tumours. Additionally, FABP4+ Mϕ showed higher abundance in adjacent non‐tumour tissues than in tumour tissues from MPLCs and SPLCs, suggesting a potential role for FABP4+ Mϕ in maintaining tissue homeostasis or exerting anti‐tumour effects in the adjacent non‐tumour tissue microenvironment.

To further substantiate the specific enrichment of *F13A1*‐ and *CD1C*‐expressing myeloid subsets in multiple primary tumours compared to normal tissues and single‐primary tumours, we firstly performed differential gene expression analysis between myeloid cells from each lesion and its corresponding adjacent non‐tumour tissue in each MPLC patient. We focused on genes that were consistently up‐regulated in both lesions. Notably, *F13A1* and *CD1C* displayed a robust and statistically significant up‐regulation in all lesions across patients with MPLCs, compared to their surrounding healthy tissues (Figure 3F and Table S8). Additionally, we conducted an in‐depth assessment of *CD1C* and *F13A1* expression levels on myeloid cells across four distinct groups and compared myeloid cells in tumour tissues from MPLCs and SPLCs. The results unequivocally demonstrated that *CD1C* and *F13A1* were significantly up‐regulated in multiple primary tumours compared to single‐primary tumours (Figure 3G,H). Moreover, their expression in adjacent non‐tumour tissues was markedly lower. The consistent up‐regulation of *F13A1* and *CD1C* across multiple nodules in different patients underscores their significance in the context of multiple primary tumours. The differential expression of these two genes between MPLCs and SPLCs further emphasises their potential as reliable markers for identifying multiple primary tumours.

### 3.5 Characterisation of dendritic cells and neutrophils in MPLCs

Given the significant enrichment of F13A1+ macrophages and CD1C+ cDC2 cells in MPLCs, we further investigated the characteristics and roles of DCs within the TME. To study the differences between tumour‐infiltrated DCs between MPLCs and SPLCs, we conducted differential analysis between these two groups. The results indicated that DCs in multi‐primary tumours exhibit high expression of major histocompatibility complex (MHC) class II molecules (*HLA‐DRB6*, *HLA‐DPB1*, *HLA‐DQB1* and *HLA‐DQA2*), as well as certain chemokines and inflammatory factors (*CXCR4*, *CXCL8*, *CXCL2*, *CXCL16*, *CCL4*, *CCL3L1*, *CCL3*, *CCL18* and *CCL17*; Figure S5A and Table S9). Conversely, DCs in solitary primary tumours showed high expression of MHC class I molecules (*HLA‐F*, *HLA‐E*, *HLA‐C*, *HLA‐B*, *HLA‐A*). GSEA demonstrated that genes up‐regulated in DCs within MPLCs were enriched for the MHC Class II‐restricted antigen presentation pathway (Figure S5B and Table S5). Cell communication analysis also revealed specific interactions between CD1C+ cDC2 cells and T/NK cells in multi‐primary tumours, highlighting the involvement of *CLEC2B/CLEC2C* molecules and *KLRB1* as ligands in this interaction (Figure S5C). This interaction is crucial as *CLEC‐2* is essential for DC migration and initiation of the cellular immune response^64^, ^65^ Additionally, *CLEC‐2* plays vital roles in thrombosis/haemostasis, tumour metastasis and lymphangiogenesis.^66^, ^67^ These findings suggested that cDC2 cells in MPLCs may have pro‐tumourigenic properties.

To further elucidate this, we focused subsequent analyses on the cDC2 cell subset, comparing differential gene expression between MPLC and SPLC groups (Table S10). GO enrichment analysis identified the top 10 GO terms, indicating that genes highly expressed in MPLC cDC2s are involved in pathways related to chemokine/cytokine‐mediated signalling, neutrophil chemotaxis and migration, response to interleukin‐1 and inflammatory response (Figure S5D). Specifically, chemokines and cytokines associated with these pathways include *CCL17*, *CXCL2*, *CXCL8*, *CCL3*, *CCL4* and *IL1B* that have been reported to negatively regulate the immune system and promote tumour development (Table S11).^68^, ^69^, ^70^, ^71^, ^72^, ^73^, ^74^, ^75^

As cDC2 cells could influence neutrophil chemotaxis and migration, we examined neutrophil gene expression and found that neutrophils in multi‐primary tumours exhibited high expression of *LYZ*, *TNFAIP3*, *G0S2*, *NFKBIA*, *C5AR1*, *PDE4B* and *FGL2* (Figure S5E and Table S12), which have been reported to inhibit anti‐inflammatory potential and promote the formation of neutrophil extracellular traps (NETs).^76^, ^77^, ^78^, ^79^, ^80^, ^81^ GSEA also showed significantly higher NET activity in neutrophils from MPLCs compared to SPLCs (NES = 4.29, adjusted *p* value <.01; Figure S5F and Table S5). Furthermore, an intriguing observation in multi‐primary tumours was the enhanced interaction between *CXCL2/3/5/8* in macrophages and *CXCR1/2* in neutrophils (Figure S5G) that has been previously reported to play critical roles in forming NETs in human neutrophils.^80^ Notably, NETs have been shown to protect cancer cells from cytotoxic actions of immune cells, particularly CD8+ T cells and NK cells, potentially promoting tumour growth. These findings highlight the potential of targeting NETs and associated pathways in the management of unresectable MPLCs.

### 3.6 F13A1+ Mϕ resembles the M2‐like macrophage

**FIGURE 4:**
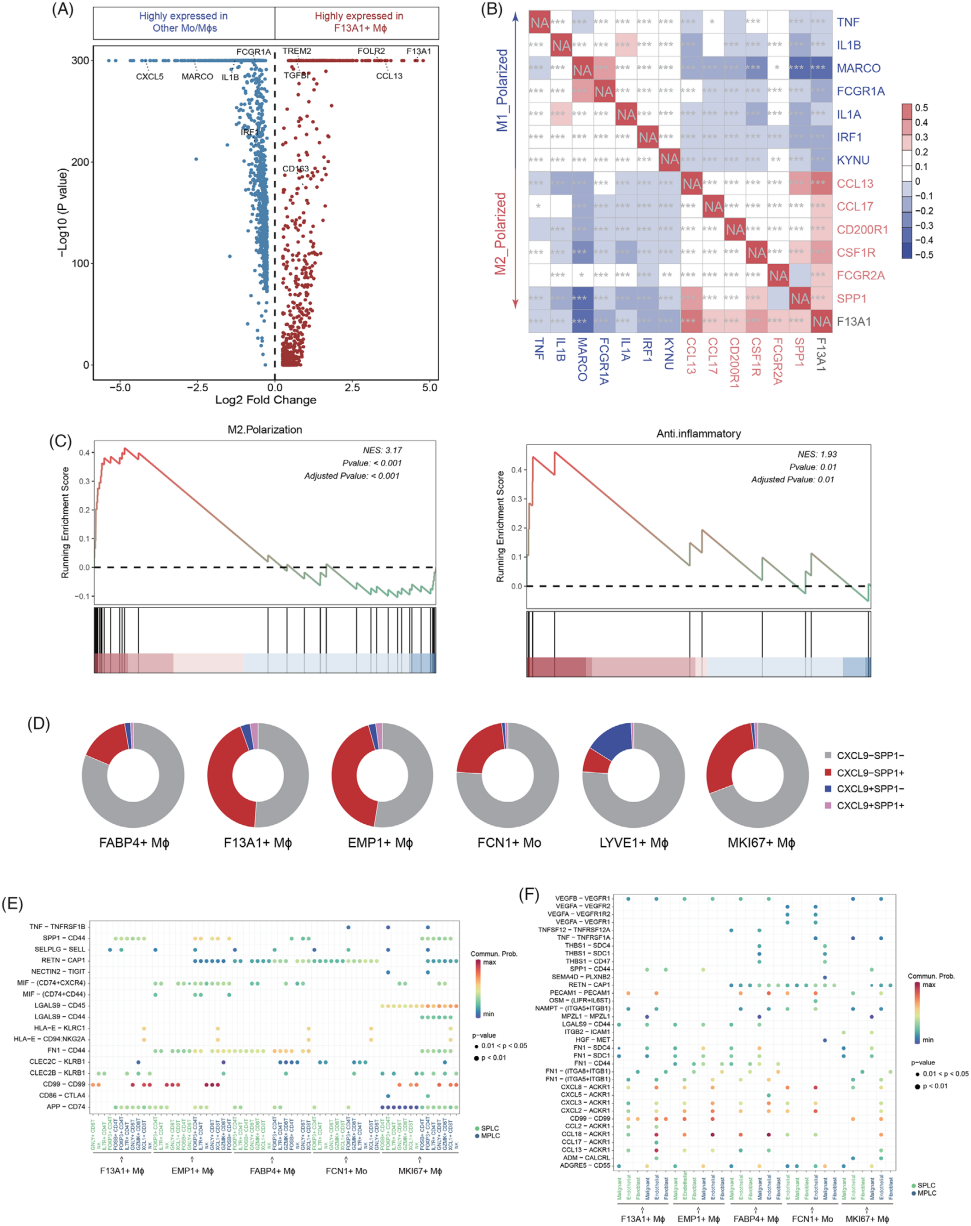
F13A1+ Mϕ resembles the M2‐like macrophage. (A) Volcano plot showing differentially expressed genes for cells from F13A1+ Mϕ and other Mo/Mϕ populations. Genes highly expressed in F13A1+ Mϕ are shown in red, and those highly expressed in other Mo/Mϕs are shown in blue. (B) Heatmap of Spearman's rank correlation between *F13A1* and markers for pro‐inflammatory M1 macrophages (in blue) and anti‐inflammatory M2 macrophages (in red), ^***^ *p* < .001. (C) Gene Set Enrichment Analysis (GSEA) analysis demonstrating that genes with higher expression in F13A1+ Mϕ compared to other Mo/Mϕs are enriched in M2 polarisation and anti‐inflammatory pathway. (D) Pie charts show the relative distribution of CXCL9−SPP1−, CXCL9−SPP1+, CXCL9+SPP1− and CXCL9+SPP1+ Mo/Mϕs in the six Mo/Mϕ cell types (FABP4+ Mϕ, F13A1+ Mϕ, EMP1+ Mϕ, FCN1+ Mo, LYVE1+ Mϕ and MKI67+ Mϕ). (E) Dot plots derived from CellChat analysis showing ligand–receptor interactions between Mo/Mϕs and T/NK cells. The size of the circles represents the *p* values, and the colour indicates the interaction strength of interacting pairs. The interactions labelled blue and red on the *x*‐axis indicate that they act as receptor‐ligand pairs in the MPLCs and SPLCs respectively. (F) Dot plot from CellChat analysis showing ligand–receptor interactions between Mo/Mϕs and endothelial cells, fibroblasts and malignant cells. The size of the circles represents *p* values, and the colour indicates the mean average expression level of interacting pairs. Interactions are labelled blue and red on the *x*‐axis to indicate MPLCs and SPLCs, respectively.

Next we focused on the F13A1+ Mϕ, the other population specifically enriched in MPLC, to further elucidate their distinct characteristics and roles in the TME of multiple primary tumours. Differential gene expression analysis comparing F13A1+ Mϕ with other Mo/Mϕ populations revealed an up‐regulation of M2 (anti‐inflammatory) macrophage markers, such as *SPP1*, *CCL13*, *FCGR2A* and *CCL18*, and a down‐regulation of M1 (pro‐inflammatory) macrophage markers, including *MACRO*, *IL1B*, *FCGR1A*, *CXCL16* and *IRF1* (Figure 4A and Table S13). A gene co‐expression analysis based on Spearman's correlation was performed to assess the functional implications of F13A1+ Mϕ by comparing their marker genes with functionally annotated signature genes. The results revealed a positive correlation between F13A1 transcript levels and several M2‐type markers, while a negative correlation was observed with M1‐type markers (Figure 4B). Consistent with these findings, GSEA showed an enrichment of anti‐inflammatory and M2 polarisation pathways in F13A1+ Mϕ over other Mo/Mϕ subsets (Figure 4C and Table S5).

In addition to the M1 versus M2 phenotyping, an alternative classification method using the relative expression levels of *CXCL9* and *SPP1* has been introduced to assess macrophage polarity, showing that the CXCL9:SPP1 ratio is associated with prognosis in cancer patients.^82^ Inspired by this, we categorised Mo/Mϕ subsets based on their expression of *CXCL9* and *SPP1*, and revealed that F13A1+ Mϕ had the highest proportion of SPP1+ cells (45.82%; Figure 4D). Additionally, CXCL9‐SPP1+ cells comprised 43.26% of the overall F13A1+ Mϕ population, suggesting a relatively low CXCL9:SPP1 ratio. This skewed ratio is indicative of a pro‐tumour effect, further highlighting the role of F13A1+ Mϕ in shaping the TME. Although the EMP1+ Mϕ subset exhibited an M2‐skewed phenotype similar to F13A1+ Mϕ, it did not show specific enrichment in MPLC (Figure 4D). This suggests that while both subsets display M2‐like polarisation, F13A1+ Mϕ plays a more specialised role in the tumour progression in MPLC.

To elucidate the interactions involving F13A1+ Mϕ and their role in the multi‐primary TME, we investigated the intercellular communications between Mo/Mϕs and T cells in MPLCs and SPLCs. Analysis of myeloid–T cell interplay showed that *HLA‐E/KLRC1*, *HLA‐E/CD94:NKG2A*‐mediated crosstalk between Mo/Mϕs and XCL1+ CD8T cells were exclusively identified in MPLCs (Figure 4E). The dimerisation of *NKG2A* (*KLRC1*) with *CD94* has been reported as a potent immune checkpoint that mediates an inhibitory signal to the NK or CD8+T cells.^83^, ^84^, ^85^ Meanwhile, the *SPP1‐CD44* interaction between myeloid and T cells was uniquely detected in MPLCs as opposed to SPLCs. Among myeloid subsets, F13A1+ Mϕ and EMP1+ Mϕ, exhibiting M2‐like polarisation, showed the strongest interaction with T cells within MPLCs, indicating that these macrophages may exert their suppressive effects on T cell function mainly through the *SPP1‐CD44* axis. As for communications between myeloid subsets and other cell types, we observed that the intensity of the interaction between *CXCL2/CXCL3/CXCL5/CXCL8/CCL17/CCL13* and *ACKR1* ligands was stronger between Mo/Mϕs and endothelial cells within MPLCs compared to SPLCs (Figure 4F). Notably, the interaction of *CCL13/CCL17* with endothelial cells was strongest in the F13A1+ Mϕ subset compared to other macrophage subtypes. *CCL13* and *CCL17* are chemokines secreted by anti‐inflammatory M2 macrophage, which are associated with pro‐angiogenic effects, promoting endothelial cell migration, invasion and proliferation through several different mechanisms.^86^, ^87^, ^88^, ^89^ *ACKR1* on the surface of endothelial cells binds, clears and regulates various chemokines such as *CXCL1*, *CXCL2*, *CCL13* and *CCL18*, which play key roles in angiogenesis and tumour progression.^90^ By clearing these chemokines, *ACKR1* can reduce their concentration in the TME, thereby decreasing the attraction and migration of tumour cells and inhibiting tumour growth and spread. This may contribute to preventing the metastasis of multi‐primary tumour cell in MPLCs.

### 3.7 Differentiation and prognostic impact of F13A1+ macrophages in MPLCs

**FIGURE 5:**
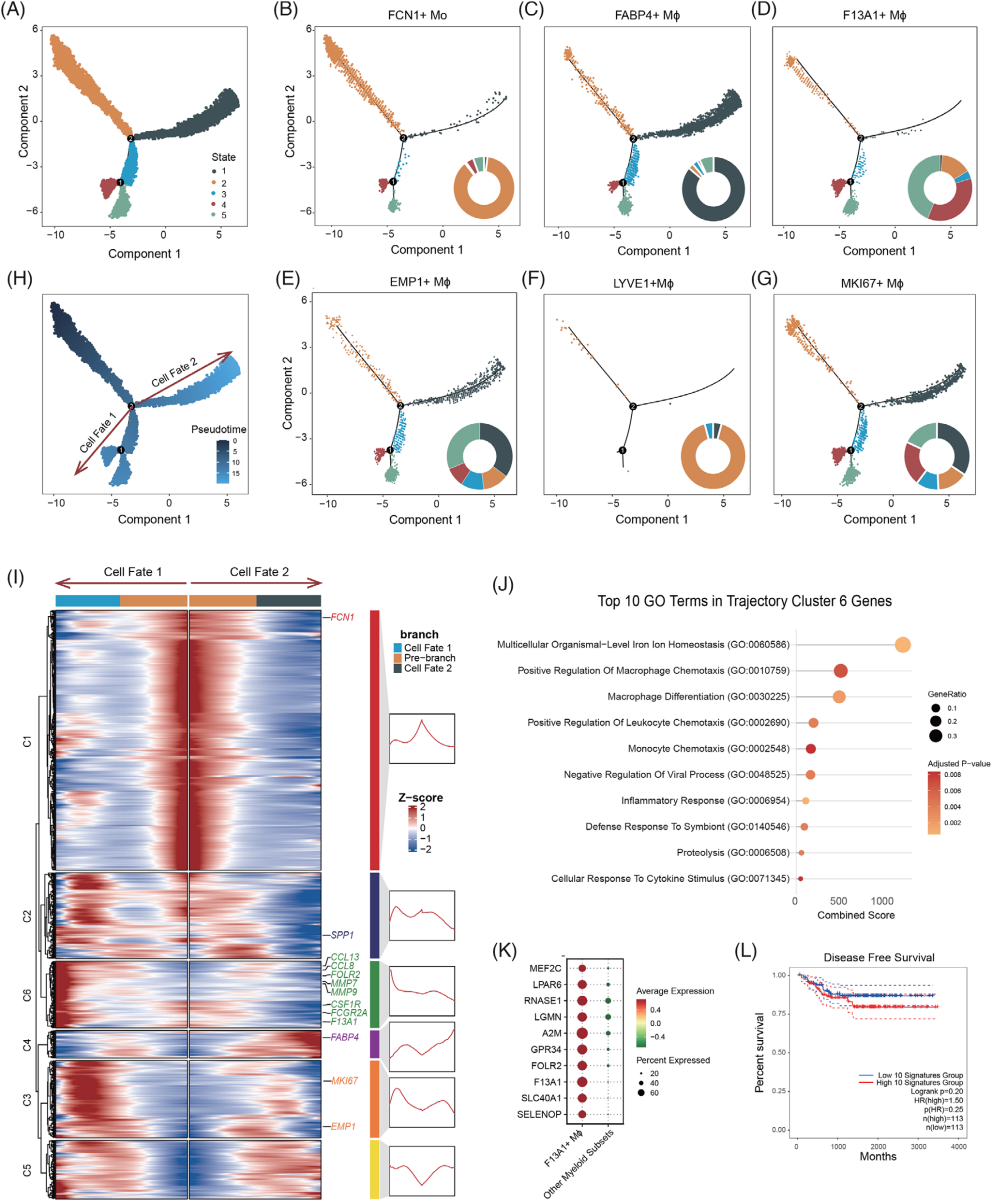
Trajectory analysis of monocyte and macrophages with monocle2 illustrates the kinetics of gene modules that are differentially expressed across the development trajectory. (A) State plot of all myeloid cells showing their differentiation trajectory. Each colour represents a different state. (B–G) Distribution of six Mo/Mϕ subgroups (FCN1+ Mo, FABP4+ Mϕ, F13A1+ Mϕ, EMP1+ Mϕ, LYVE1+ Mϕ and MKI67+ Mϕ). Each plot highlights the specific subgroup, and the accompanying pie charts show the proportion of cells in each state for the respective subgroup. (H) Pseudotime plot illustrating the progression of cells along the differentiation trajectory, highlighting two distinct cell fates (Cell Fate 1 and Cell Fate 2). (I) Branched kinetic headman showing the expression clustering of the top 1000 genes during Mo/Mϕ differentiation. Genes (*row*) are clustered and cells (*column*) are ordered according to the pseudotime development along the two cell fates. (J) Gene ontology (GO) analysis of genes in Cluster 6, indicating enrichment in positive regulation of macrophage chemotaxis (GO:0010759), macrophage differentiation (GO:0030225) and monocyte chemotaxis (GO:0002548). (K) Dot plot showing the top 10 differentially expressed marker genes between F13A1+ Mϕ and other myeloid groups, ranked by log2FC. The size of the dots represents the fraction of cells expressing a particular marker, and the colour intensity indicates the average expression level. (L) Kaplan–Meier survival curves showing the disease‐free survival (DFS) of lung cancer patients in the GSE31210 dataset, stratified by the expression of F13A1+Mϕ marker genes. The analysis was conducted with the cutoff set at the median expression level. The curves compare patients with high and low expression of these markers, illustrating their impact on disease progression and patient outcomes.

Having established the pro‐tumourigenesis and anti‐immune features of F13A1+ Mϕ, we moved to dissect the developmental origin of these cells. We conducted a comprehensive trajectory analysis on Mo/Mϕ and investigated gene expression dynamics along the differentiation pathway. Trajectory analysis of Mo/Mϕ cells identified five distinct states (Figure 5A). The early macrophage‐specific gene *CLEC4E* was significantly expressed in state 2 (Figure S6A), suggesting that state 2 is the starting point of differentiation. Examination of the six monocyte/macrophage subgroups showed that they are located at different developmental states (Figure 5B–G). FCN1+ Mo cells are primarily located in state 2, indicating that these cells are the differentiation starting point and they could be the origin of other Mϕ subtypes (Figure 5B). The majority of FABP4+ Mϕ cells are in state 1, while a significant portion of F13A1+ Mϕ cells are in states 4 and 5 at the opposite differentiation branch, suggesting these two subsets represent two Mϕ sub‐populations that are generated via distinct cellular pathways (Figure 5C,D). EMP1+ Mϕ cells are predominantly located in state 1 and state 5 (Figure 5E), whereas LYVE1+ subset consists of only few cells, mostly located in state 2 (Figure 5F). Unlike other subsets, MKI67+ Mϕ cells are evenly distributed across all cellular states, and may not associated with a specific differentiation program (Figure 5G).

To infer the dynamic transcriptional programs along different macrophage trajectories, we first identified two distinct cell fates via pseudotime analysis. Cell Fate 1 involves the differentiation of FCN1+ Mo to F13A1+ Mϕ, while Cell Fate 2 involves the differentiation of FCN1+ Mo to FABP4+ Mϕ (Figure 5H). Top 1000 genes that show most variable expression patterns between these two pathways were clustered into six modules (Figure 5I and Table S14). Cluster 1 (C1) genes, including *FCN1*, were highly expressed at the early differentiation stage, in line with the observation that FCN1+ Mo cells are located at the differentiation starting point. Cluster 4 (C4) genes, including *FABP4*, were highly expressed at the end of Cell Fate 2, and are associated with FABP4+ Mϕ differentiation. Cluster 6 (C6) genes, including *CCL13*, *CCL8*, *FOLR2*, *MMP7*, *MMP9*, *CSF1R* and *FCGR2A*, exhibited increased expression as differentiation progressed, particularly at the end of the Cell Fate 1 differentiation trajectory. Notably, these genes have been documented in the literature to be associated with macrophage polarisation towards the M2 phenotype.^91^, ^92^, ^93^, ^94^, ^95^, ^96^, ^97^, ^98^ GO analysis of C6 genes showed significant enrichment of transcriptional programs associated with macrophage differentiation, positive regulation of macrophage chemotaxis and monocyte chemotaxis (Figure 5J and Table S15). These are consistent with the observation that F13A1+ Mϕs are located at a later stage of the differentiation trajectory, indicative of a terminally differentiated phenotype.

To further validate the differentiation trajectories identified by Monocle2, we applied Slingshot analysis to monocyte and macrophage populations. Since MKI67+ macrophages represent proliferative cells spread across multiple differentiation states, their inclusion could obscure trajectory inference. Thus, we excluded MKI67+ macrophages to achieve clearer differentiation pathways. Slingshot analysis revealed four distinct lineages (Figure S6B,C). The differentiation paths from FCN1+ Mo to FABP4+ Mϕ (Lineage 3) and from FCN1+ Mo to F13A1+ Mϕ (Lineage 2) closely aligned with the major cell fates identified by Monocle2, while also revealing two additional branches: FCN1+ Mo to EMP1+ Mϕ and FCN1+ Mo to LYVE1+ Mϕ, likely reflecting subtle differences between the tools. Despite these minor discrepancies, both methods consistently highlight the key differentiation pathways, particularly the terminal differentiation of F13A1+ Mϕ.

Given the potential clinical significance of F13A1+ Mϕ as terminally differentiated M2‐type macrophages in MPLC, we further explored their prognostic impact. We analysed gene expression profiles between F13A1+ macrophages and other myeloid subtypes to explore the clinical relevance and prognostic impact of these terminal/mature M2‐type macrophages that are specific to MPLC. Top 10 DEGs that are up‐regulated in F13A1+ Mϕ, ranked by log2FC, were selected as a candidate molecular signature (Figure 5K and Table S13). We examined the expression level of this 10‐gene signature in patients from GSE31210 dataset to explore the prognostic potential of this signature. Kaplan–Meier survival analysis showed that patients with high signature levels had relatively shorter DFS, with a HR of 1.5. However, the *p* value of  .25 from the Cox model was not statistically significant, and the log‐rank test also indicated no significant survival difference (*p* = .20; Figure 5L). Additionally, the prognostic impact of the F13A1+ macrophage fraction was not significant, as Kaplan–Meier analysis indicated no substantial DFS difference between high and low fractions, with a HR of 1.22 (*p* = .56, Cox model) and a log‐rank *p* value of  .60 (Figure S6D).

Overall, these findings suggest that although most macrophage subsets originate from FCN1+ monocytes, the F13A1+ Mϕs are terminally differentiated and represent a distinct cellular fate. This specific differentiation process may contribute to the formation of a TME that is unique and critical to the development of MPLC. Moreover, the 10‐gene signature derived from F13A1+ Mϕs was significantly associated with shorter DFS, highlighting its potential as a prognostic biomarker.

### 3.8 Validation of MPLC‐specific F13A1+ Mϕ and CD1C+ cDC2

**FIGURE 6:**
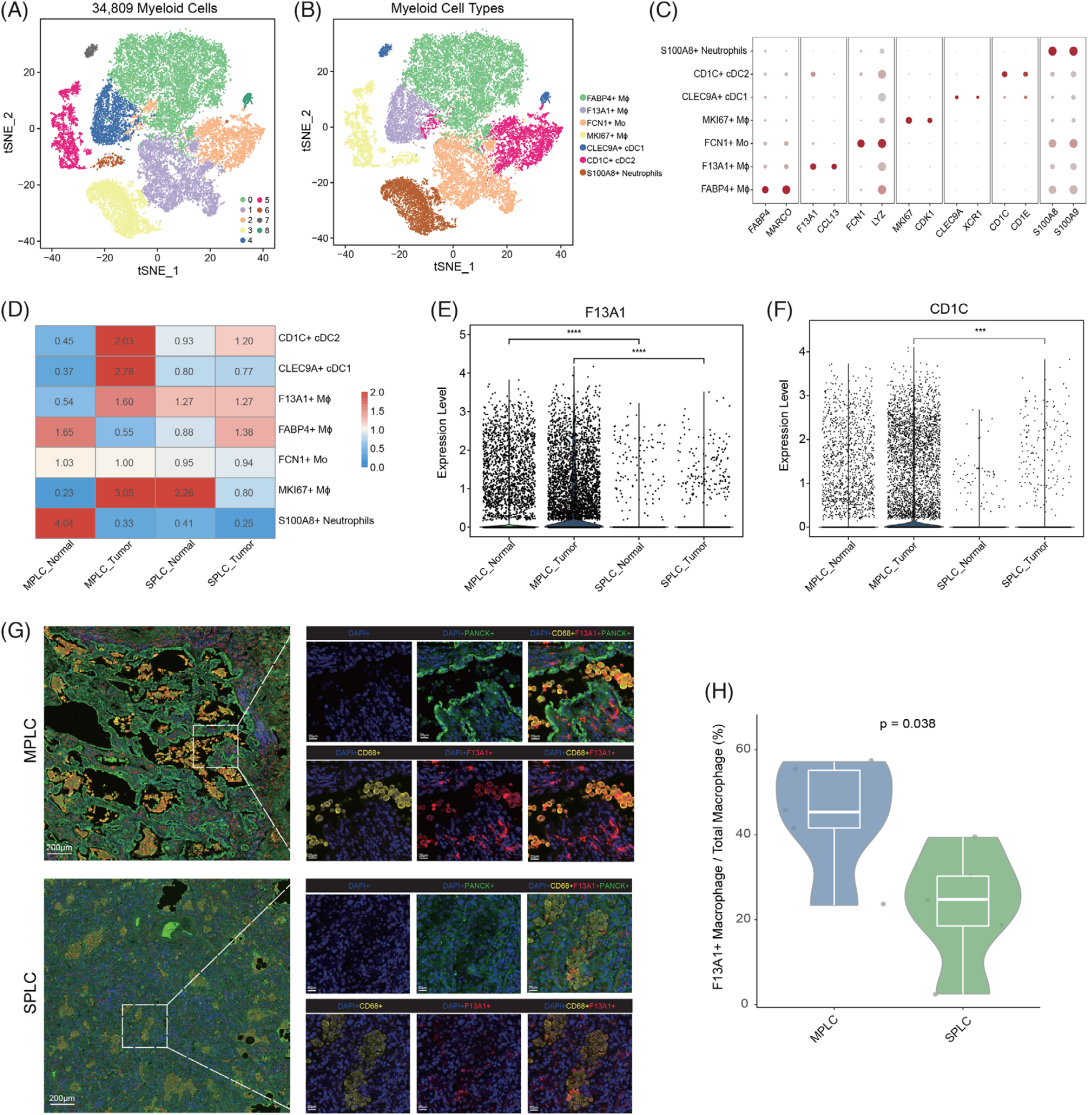
Validation of findings in external datasets and multiplex immunofluorescence. (A) t‐Distributed stochastic neighbour embedding (t‐SNE) plot showing the clustering of 34 809 myeloid cells from external datasets (GSE200972, E‐MTAB‐6149 and E‐MTAB‐6653) of MPLCs and SPLCs. Each colour represents a different cluster. (B) t‐SNE projection of myeloid cell types from the external data, annotated with the inferred cell types. (C) Dot plot displaying selected cell type‐specific markers across all myeloid cell types. The dot size represents the fraction of cells expressing a particular marker, and the colour intensity indicates the average expression level. (D) Heatmap showing the odds ratios of myeloid cell subtypes in each group. An odds ratio (OR) >1.5 indicates that the meta‐cluster is preferred to distribute in the corresponding group. (E) Violin plot showing the expression levels of *F13A1* in macrophages across the four groups, with statistical significance between MPLC_Tumour and SPLC_Tumour determined by Wilcoxon rank‐sum test. (F) Violin plot showing the expression levels of *CD1C* in dendritic cells (DCs) across the four groups, with statistical significance between MPLC_Tumour and SPLC_Tumour determined by Wilcoxon rank‐sum test. (G) Representative multiplex immunofluorescence images demonstrating the protein expression of *CD68* (yellow), *F13A1* (magenta), 4′,6‐diamidino‐2‐phenylindole (DAPI; blue) and pan‐cytokeratin (green) in MPLC (upper panel) and SPLC (lower panel) samples. Nuclei are stained with DAPI. Scale bars = 200 µm. (H) Boxplot illustrating the fraction of F13A1+ CD68+ cells (F13A1+ macrophages) out of the total macrophage population in MPLCs and SPLCs. The boxplot shows the medians, first and third quartiles, and minimum and maximum values within 1.5× interquartile range (IQR) of the box limits. Statistical significance was determined using the Wilcoxon rank‐sum test (*p* value = .038).

We analysed gene expression data from external MPLC and SPLC cohorts to independently validate our findings of MPLC‐specific myeloid sub‐populations. Publicly available scRNA‐seq data from GSE200972,^99^ E‐MTAB‐6149^100^ and E‐MTAB‐6653,^100^ comprising 25 samples from four MPLC and three SPLC patients, were included. The clinical information and sample metadata for these external cohorts are presented in Table S16. In these datasets, a total of 34 809 myeloid cells were annotated, and further classified into seven clusters, defined as FABP4+ Mϕ, F13A1+ Mϕ, FCN1+ Mo, MKI67+ Mϕ, CLEC9A+ cDC1, CD1C+ cDC2 and S100A8+ neutrophils (Figures 6A–C and S7A–C). We assessed the distribution pattern of myeloid subsets in the same manner as in our own data, and identified the enrichment of F13A1+ Mϕ (OR = 1.60) and CD1C+ cDC2 (OR = 2.03) in multi‐primary tumours compared to solitary primary tumours and adjacent non‐tumour tissues (Figure 6D). Furthermore, the expression levels of *F13A1* and *CD1C* in myeloid cells were significantly higher in MPLC tumours than SPLC tumours (Figure 6E,F). These findings are consistent with our study, reinforcing the validity and reliability of our research methodology.

We next validated the interactions of macrophages with other subsets in external datasets. The SPP1 signalling pathway (e.g., *SPP1‐CD44*, *SPP1‐*Integrin pairs such as *ITGA4+ITGB1*, *ITGA5+ITGB1* and so on)) demonstrated stronger interactions between myeloid cells and other cell types in MPLCs compared to SPLCs, further supporting our hypothesis of a distinct TME in MPLCs. Notably, the interaction of *CCL13‐ACKR1* with endothelial cells was also confirmed in the validation datasets as being specific to the F13A1+ Mϕ subset, distinguishing it from other macrophage subtypes (Figure S7D).

To further substantiate these results, we conducted multiplex immunofluorescence (mIF) staining on tumour tissues from five MPLC patients and five SPLC patients. This analysis demonstrated that the proportion of F13A1+ Mϕ (F13A1^+^ CD68^+^) within the total CD68^+^ macrophage population was significantly higher in MPLC samples compared to SPLC samples. Statistical comparison using the Wilcoxon rank‐sum test confirmed this finding (*p* value = .038), in line with the differential distribution patterns of F13A1+ Mϕ observed in the scRNA‐seq analysis (Figure 6G,H).

Taken together, these results across different datasets and studies consistently demonstrate the potential of *F13A1* and *CD1C* as biomarkers for distinguishing between MPLCs and SPLCs. The identification of these specific markers in MPLC tumours offers a promising venue for targeted therapeutic interventions and could pave the way for more personalised treatment approaches in MPLC management.

## 4 DISCUSSION

The prevalence of MPLCs is continuously rising. However, the establishment of a standardised guideline and determination of optimal diagnostic and therapeutic strategies are still hindered by the lack of sufficient evidence, leading to on‐going scientific debates in this field.

Previous studies on MPLCs have significantly advanced our understanding of this complex disease. Many of these studies have focused on comparing MPLC tumours with adjacent normal tissues or analysing different stages of MPLC tumour tissues (from normal samples to adenocarcinoma in situ [AIS], minimally invasive adenocarcinoma [MIA] and ultimately invasive adenocarcinoma [IAC]), to elucidate the changes in the TME during disease progression. These studies provide a comprehensive summary of the dynamic changes in cellular composition and gene activity that occur throughout disease progression.^18^, ^19^ Some studies explored the heterogeneity among tumour lesions within the same MPLC patient, revealing that these lesions possess distinct genetic profiles and cellular compositions resulting in varied responses to treatment, which further complicates the clinical practice.^19^, ^99^ Dissection of the genetic and cellular characteristic of MPLC and intrapulmonary metastasis (IPM) have discovered a novel epithelial sub‐population referred to as CLDN2+ alveolar type II (AT2) cells.^99^ These cells are specifically enriched in MPLC and play significant roles in cellular communication and tumour progression, facilitating more accurate diagnoses and tailored therapies. The expression of *CLDN2* could help distinguish MPLC from IPM, but the underlying mechanism driving the emergence of CLDN2+ AT2 cells in MPLC requires further experimental investigation.^99^

In our study, instead of incorporating various individual public MPLC and SPLC datasets, we performed a comparative scRNA‐seq analysis to simultaneously profile tumour and adjacent paratumour samples from both MPLC and SPLC patients. This introduces several novel aspects in experimental design compared to previous studies. Our study also offers a perspective that diverges from previous studies that primarily focused on intra‐MPLC comparisons or distinguishing MPLC from IPM. We found that MPLC and SPLC shared significant similarities in various aspects, including structural genomic variants, driver gene mutations, transcriptomic alterations, cellular processes, signalling pathways and immune checkpoint expressions, strongly suggesting a substantial degree of resemblance between the two subtypes. The extensive research on SPLCs provides a wealth of knowledge that can inform and guide future studies on MPLCs. Our data, consistent with previous studies, showed a high prevalence of oncogenic driver mutations, such as EGFR mutations, in both MPLC and SPLC patients.^101^ While several studies have documented the effectiveness of EGFR‐TKI therapy in SPLC patients,^102^, ^103^, ^104^ there is a noticeable scarcity of research focusing specifically on the efficacy of EGFR‐TKI treatment in MPLCs. The frequent occurrence of EGFR mutations in both subtypes suggests that EGFR‐TKIs could be a viable treatment options for MPLC patients as well. Additional research is required to evaluate the response rates and therapeutic benefits of EGFR‐TKIs specifically in the context of MPLCs.

The landscape of tumour‐infiltrating T/NK cell populations also displays great similarities between both tumour types. Several studies have established that various cancers carry a large number of tumour‐infiltrating Treg cells,^105^, ^106^, ^107^, ^108^ and the abundance of naïve CD4+ T cells and Tregs in cancer patients has been found to be closely associated with poor prognosis.^109^, ^110^ Although both T cell populations are enriched MPLC and SPLC tumours, there is no difference between them. Furthermore, the expression levels of ICGs and exhaustion markers, such as *PD‐1* and *TIGIT*, are at comparable levels between the two tumour types, suggesting that existing immunotherapy targeting PD‐1/PD‐L1 may yield equivalent efficacy for both tumour types. As current studies applying ICIs do not distinguish between MPLCs and SPLCs, it remains largely unknown if patients with these two cancer types exhibit distinct response to ICI treatments. Direct comparisons between MPLCs and SPLCs may provide valuable insights into the response patterns and treatment effectiveness of ICIs in different tumour subtypes.

Recent studies have revealed the multifaceted role of neutrophils in lung cancers. In our study, a specific enrichment of the NETosis pathway in neutrophils from MPLCs compared to SPLCs was observed. These neutrophils engaged with other cell types through *CXCR1/2*‐related ligands. NETs are web‐like structures formed by dying neutrophils that extrude chromatin into the extracellular space, and play an important role in carcinogenesis, both by triggering DNA damage indirectly through inflammation and by directly promoting a pro‐tumour TME.^111^, ^112^ Inhibition of NETs and NETosis using DNAse I treatment and peptidylarginine deiminase (PAD) inhibitors interfere has shown great potential as a therapeutic option for treating cancer.^113^, ^114^ The C‐X‐C chemokine receptor (CXCR) signalling pathways, which play an important role in neutrophil chemotaxis as well as in NET formation,^115^ is another group of promising targets for mitigating the pro‐tumour effects of neutrophils. Several *CXCR1* and *CXCR2* inhibitors, including Navarixin and SX‐682, are currently under investigation for their efficacies in various solid tumours (NCT03473925 and NCT04477343). These findings highlight the potential of targeting the *CXCR1/2* pathways and NETosis in MPLC therapy.

The identification of the unique F13A1+ Mϕ subtype and its preferential enrichment in MPLC is a significant discovery in this study. The overexpression of *F13A1* in tumour‐infiltrating Mo/Mϕs compared to adjacent non‐cancerous tissues suggests its potential as a diagnostic and prognostic marker for MPLC. The enrichment of F13A1+ Mϕs in MPLCs compared to SPLCs suggests their involvement in the formation of TME, as well as the distinct characteristics and behaviour of MPLC tumours, potentially linked to immune evasion mechanisms and a tumour‐promoting phenotype specific in MPLC. At transcriptional levels, our data show that F13A1+ Mϕs resemble the M2‐like macrophages, an anti‐inflammatory subtype that has been implicated in promoting tumour growth and suppressing immune response.^116^ Cell–cell communication analysis suggests a crucial role of F13A1+ Mϕs in the crosstalk with other cells in TME through specific ligand–receptor interactions. The *SPP1‐CD44* axis emerges as the predominant communication route between F13A1+ Mϕs and other cells in MPLCs, while the *CCL13‐ACKR1* interaction with endothelial cells is preferentially elevated in F13A1+ Mϕs compared to other macrophage subtypes in MPLC. *SPP1*, also known as osteopontin, is expressed by various cell types, including tumour‐associated macrophages, and its high expression on TAMs often indicates negative prognosis and chemoresistance in lung cancer.^117^ Additionally, the *SPP1‐CD44* axis has been implicated in cancer chemoresistance by impacting the pro‐tumour function of TAMs.^118^ *CCL13*, a chemokine secreted by anti‐inflammatory M2 macrophages, is associated with pro‐angiogenic effects, promoting endothelial cell migration, invasion and proliferation through several different mechanisms.^86^ However, the role of *SPP1‐CD44* and *CCL13‐ACKR1* interactions has not been well characterised in the context of MPLC. Our findings that both *SPP1* and *CCL13*, along with their respective interaction, are up‐regulated in F13A1+ Mϕs indicate their involvement in shaping the immunosuppressive TME, facilitating tumour immune escape and promoting tumour proliferation and invasion in MPLC.

It is worth noting that the enrichment of F13A1+ Mϕs in MPLC patients was also observed in independent validation datasets, providing further evidence of their preferential presence in MPLC. Additionally, mIF staining on tumour tissues from MPLC and SPLC patients further substantiate the results, strengthening our hypothesis and confirming the differential distribution patterns observed in the scRNA‐seq analysis. The presence of F13A1+ Mϕs in MPLC suggests their involvement in the formation of the TME, potentially linked to immune evasion mechanisms and a tumour‐promoting phenotype specific to MPLC. Targeting the immunosuppressive and tumour‐promoting properties of F13A1+ Mϕs could open new avenues for therapeutic interventions in MPLC.

A major limitation of our study is the need for validation in larger patient cohorts to fully establish the clinical significance of F13A1+ Mϕs as a marker for MPLC. Further investigation into the underlying mechanisms driving the emergence, expansion and functionality of F13A1+ Mϕs in MPLC is warranted to fully comprehend their molecular mechanisms, functional roles and therapeutic implications. Additionally, future work will include experimental validation of the *SPP1‐CD44* and *CCL13‐ACKR1* interaction to better understand their roles in the TME of MPLC.

In summary, this study employed scRNA‐seq to comprehensively analyse tumour and adjacent samples from both MPLCs and SPLCs. The findings unveiled notable similarities between the two tumour types, hinting at shared molecular mechanisms driving their development. Moreover, by identifying and characterising specific cell populations and unique alterations in MPLCs, this study provides valuable insights into the intricate heterogeneity and complexity of MPLCs. These results highlight the importance of future research to unravel the underlying biological processes and molecular drivers specific to MPLCs. Ultimately, gaining a deeper understanding of these distinct features holds great potential in guiding the development of personalised treatment strategies for patients with MPLCs.

## AUTHOR CONTRIBUTIONS

The authors confirm contribution to the paper as follows: Conceptualisation and Funding acquisition: Xiaotong Guo; Validation and Investigation: Chenglin Yang; Data Curation: Jingting Wu; Formal analysis: Jiahao Qu; Resources: Songhua Cai; Visualisation: Wenyi Liu, Liuqing Zheng and Lishen Zhang; Software: Liuqing Zheng and Lishen Zhang; Writing—Original Draft: Liuqing Zheng and Chenglin Yang; Writing—Review & Editing: Jiahao Qu, Li Wang and Yiran Meng; Supervision: Youjun Deng and Yiran Meng. All the authors reviewed the results and approved the final version of the manuscript.

## CONFLICT OF INTEREST STATEMENT

The authors declare no conflicts of interest.

## ETHICS STATEMENT

This study was conducted in accordance with the Declaration of Helsinki, and the protocol was approved by the Institutional Review Board of the Medical Research Ethics Committee of National Cancer Center/National Clinical Research Center for Cancer/Cancer Hospital & Shenzhen Hospital, Chinese Academy of Medical Sciences and Peking Union Medical College (approval number: KYKT2023‐32‐1). Informed consent was obtained from all individual participants included in the study.

## Supporting information



Supporting Information

**FIGURE S1** Clustering of 327 611 single cells from MPLC and SPLC patients, related to Figure 1. (A) t‐SNE plot showing the clustering of 327 611 cells profiled here, with each cell colour‐coded by its corresponding sample ID. (B) Bar plot illustrating the fractions of major cell types detected in each group, coloured‐coded by major cell lineages as shown in Figure 1. (C) t‐SNE projections within each group, colour‐coded by major cell lineages. (D) Heatmap displaying the top 10 differentially expressed genes (rows) according to the log2FC across major cell types (columns). The heatmap is organised by major cell lineages, with genes colour‐coded to indicate their relative expression levels.

**FIGURE S2** Genomic and transcriptomic analysis of epithelial cell lineages in MPLCs and SPLCs. (A) Representative CNV heatmaps obtained from inferCNV analysis grouped by tumour lesion. Each column represents a single cell, and each row represents a different chromosomal region. (B) Violin plots showing distributions of CNV scores among different groups, including epithelial cells from MPLCs, SPLCs and normal tissues. (C) Venn diagram showing the overlap of differentially expressed genes (DEGs) in malignant cells of MPLCs and SPLCs compared to epithelial cells from normal tissues. The top part of the diagram shows the overlap of up‐regulated DEGs, while the bottom part shows the overlap of down‐regulated DEGs. Thirty‐six up‐regulated and 64 down‐regulated DEGs common to both MPLCs and SPLCs. (D) Gene Set Enrichment Analysis (GSEA) analysis showing significant enrichment of genes with higher expression in malignant cells of MPLCs (top) and SPLCs (bottom) compared to epithelial cells from normal tissues. The enriched pathways include hallmarks of epithelial–mesenchymal transition (EMT), p53 pathway. (E) Stacked violin plot showing the expression levels of immune checkpoint molecules across different tumour samples. (F) Clonality trees for each of the 13 tumour lesion. The branches of the trees are scaled according to the percentage of cells present in each subclone with the corresponding CNV event. Each tree represents the clonal architecture and evolution of the tumour lesions. (G) Summary plot of the CNV events from each of the 13 tumour lesions inferred from their scRNA‐seq data. The plot shows the presence or absence of CNV events across different chromosomal regions for each tumour lesion, grouped by MPLC and SPLC.

**FIGURE S3** T/NK cell subsets, related to Figure 2. (A) t‐SNE plot illustrating the clustering of 90 152 T/NK cells, with each cell colour‐coded by its corresponding sample ID. (B) Bar plot depicting the fractions of T/NK cell types detected in each group, coloured‐coded by major T/NK cell lineages as shown in Figure 3. (C) t‐SNE projections within each group, colour‐coded by T/NK cell lineages. (D) Heatmap showing the top 10 differentially expressed genes (rows) according to the log2FC across T/NK cell types (columns). The heatmap is organised by T/NK cell lineages, with genes colour‐coded to indicate their relative expression levels. (E) Violin plots displaying the dysfunction scores of CD8+ Tex cells across different samples (M1T1–S3T). The right panel shows a box plot comparing the median T cell dysfunction scores between MPLC and SPLC groups, indicating no significant difference (ns). (F) Violin plots showing the cytotoxic scores of CD8+ Teff cells across different samples (M1T1–S3T). The right panel presents a box plot comparing the median T cell cytotoxic scores between MPLC and SPLC groups, demonstrating significantly higher scores in MPLC. (G) T cell proliferation scores: Violin plots depicting the proliferation scores of T cells across different samples (M1T1–S3T). The right panel illustrates a box plot comparing the median T cell proliferation scores between MPLC and SPLC groups, indicating no significant difference (ns).

**FIGURE S4** Myeloid cell subsets, related to Figure 3. (A) t‐SNE plot illustrating the clustering of 118 556 myeloid cells, with each cell colour‐coded by its corresponding sample ID. (B) Bar plot depicting the fractions of myeloid cell types detected in each group, coloured‐coded by major cell lineages as in Figure 4. (C) t‐SNE projection within each group, colour‐coded by myeloid cell lineages. (D) Heatmap displaying the top 10 differentially expressed genes (rows) according to the log2FC across myeloid cell types (columns). The heatmap is organised by major cell lineages, with genes colour‐coded to indicate their relative expression levels.

**FIGURE S5** Characterisation of dendritic cells (DCs) and neutrophils. (A) Volcano plot showing differentially expressed genes between DCs from MPLC and SPLC. Red dots represent up‐regulated genes in MPLC DCs, and blue dots represent up‐regulated genes in SPLC DCs. (B) Gene Set Enrichment Analysis (GSEA) indicating that genes with higher expression in DCs from MPLCs are significantly enriched in the MHC class II‐restricted antigen presentation pathway. (C) Dot plot illustrating the communication between T/NK cells and DCs in MPLC and SPLC. The size of the dots represents the *p* value of the interaction, and the colour indicates the communicating probability. (D) The bubble plot displaying the top 10 enriched GO terms in cDC2 cells from MPLC samples. The *x*‐axis represents the combined score; the *y*‐axis lists the GO terms. The size of each bubble corresponds to the GeneRatio, and the colour indicates the adjusted *p* value, with darker shades representing more significant *p* values. (E) Volcano plot illustrating differentially expressed genes between neutrophils from MPLCs and SPLCs. Genes with significantly higher expression in MPLC neutrophils are shown in red, while those with higher expression in SPLC neutrophils are shown in blue. (F) Gene Set Enrichment Analysis revealed an enrichment of genes with higher expression in neutrophils from MPLCs, compared to those from SPLCs, in the NETosis pathway. (G) Dot plot depicting the communication between neutrophils and other cell types in both MPLC and SPLC tumours. The size of the dots represents the statistical significance (*p* value), with larger dots indicating stronger significance. The colour intensity represents the communication probability.

**FIGURE S6** Trajectory and prognostic analysis of F13A1+ Mϕ. (A) Expression of the early macrophage‐specific gene CLEC4E along the Monocle2 differentiation trajectory. (B) Trajectory analysis of Mo/Mϕ populations using Slingshot, showing four predicted lineages, each represented by a distinct colour. The red trajectory corresponds to Lineage 1, which represents the transition from FCN1+ Mo to EMP1+ Mϕ. Lineage 2 is shown in blue, indicating the path from FCN1+ Mo to F13A1+ Mϕ. The green trajectory represents Lineage 3, illustrating the transition from FCN1+ Mo to FABP4+ Mϕ while Lineage 4, displayed in purple, depicts the path from FCN1+ Mo to LYVE1+ Mϕ. These trajectories capture the potential differentiation paths inferred by Slingshot. (C) Visualisation of cells along Lineage 2 (FCN1+ Mo to F13A1+ Mϕ), with cell colours representing pseudotime progression. Cells are coloured from red (early pseudotime) to blue (late pseudotime), reflecting their position along the pseudotime axis. (D) Kaplan–Meier survival curves showing the disease‐free Survival (DFS) of lung cancer patients in the GSE31210 dataset, stratified by the F13A1+ Mϕ fraction. Patients were grouped based on the median cell fraction, comparing high and low expression levels. The curves highlight the impact of F13A1+ macrophage fraction on disease progression and patient outcomes.

**FIGURE S7** Validation of findings in external datasets. (A) t‐SNE plot showing the clustering of 160 984 cells from external datasets (GSE200972, E‐MTAB‐6149 and E‐MTAB‐6653) of MPLC and SPLC. Each colour represents a different cluster. (B) t‐SNE projection of cell types from the external data, annotated with the inferred cell types. (C) Dot plot displaying selected cell type‐specific markers across all cell types. The dot size represents the fraction of cells expressing a particular marker, and the colour intensity indicates the average expression level. (D) Dot plot illustrating the communication between Mo/Mϕs and other cell types in MPLC and SPLC, based on CellChat validation in external datasets. The size of the dots represents the *p* value of the interaction, and the colour indicates the communicating probability.

## Data Availability

The data that support the findings of this study are available from the corresponding author upon reasonable request.
