# Supplementary figures and images for: Single‐cell dissection reveals immunosuppressive F13A1+ macrophage as a hallmark for multiple primary lung cancers
Source: Clin Transl Med. 2024 Nov 27;14(12):e70091. doi: 10.1002/ctm2.70091 (PMC11600049; doi:10.1002/ctm2.70091)

A

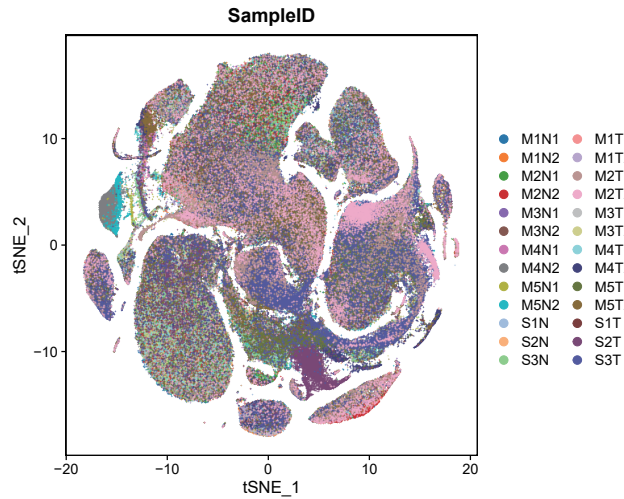

B

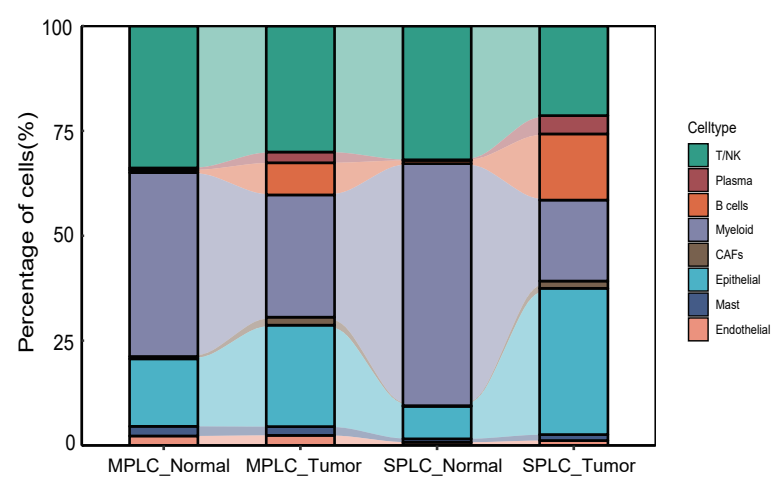

C

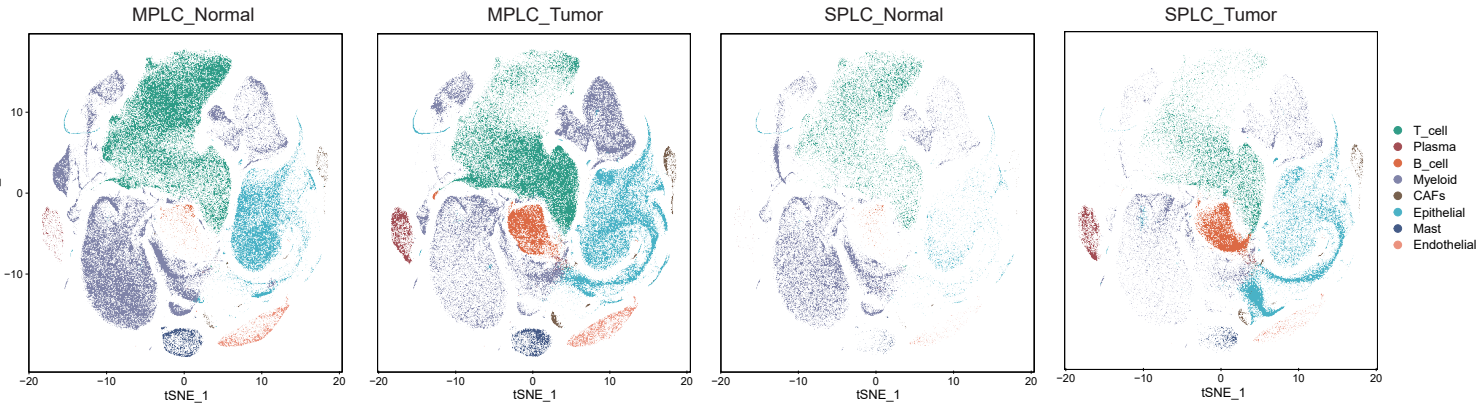

D

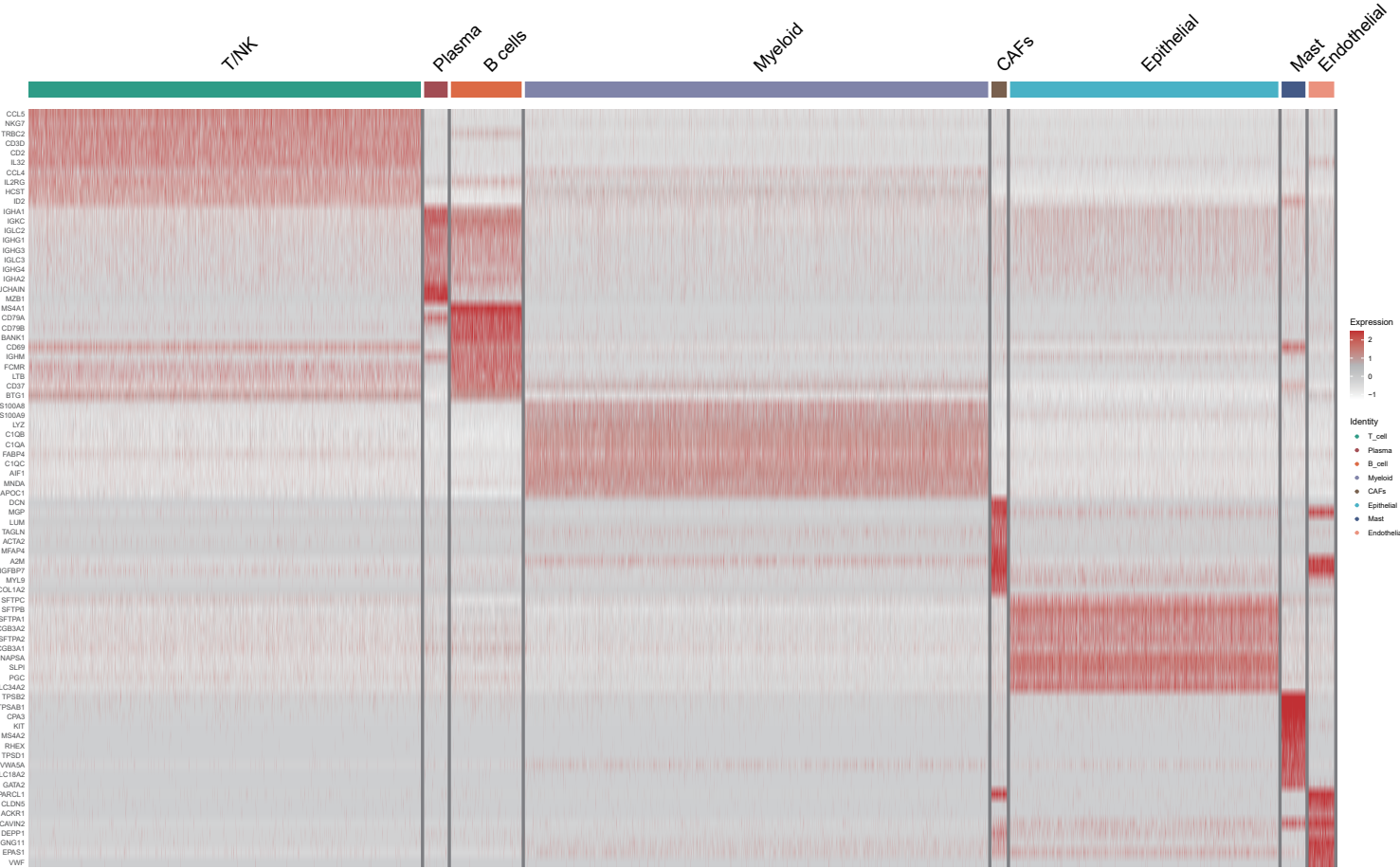

Supplement: Supplementary file 2 — FIGURE S1 Clustering of 327 611 single cells from MPLC and SPLC patients, related to Figure 1. (A) t‐SNE plot showing the clustering of 327 611 cells profiled here, with each cell colour‐coded by its corresponding sample ID. (B) Bar plot illustrating the fractions of major cell types detected in each group, coloured‐coded by major cell lineages as shown in Figure 1. (C) t‐SNE projections within each group, colour‐coded by major cell lineages. (D) Heatmap displaying the top 10 differentially expressed genes (rows) according to the log2FC across major cell types (columns). The heatmap is organised by major cell lineages, with genes colour‐coded to indicate their relative expression levels. [file CTM2-14-e70091-s001.pdf]

A

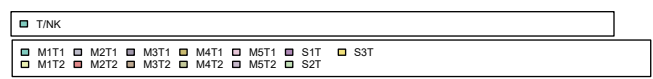

B

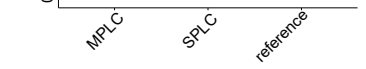

C

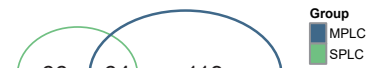

1

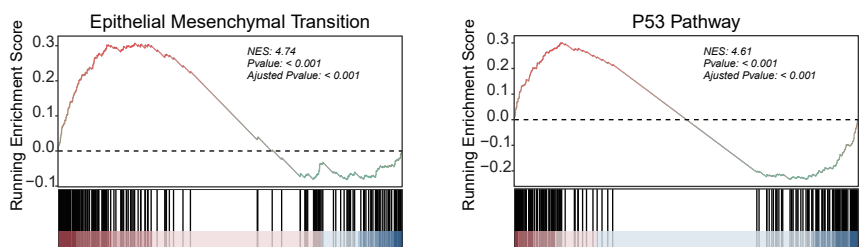

1

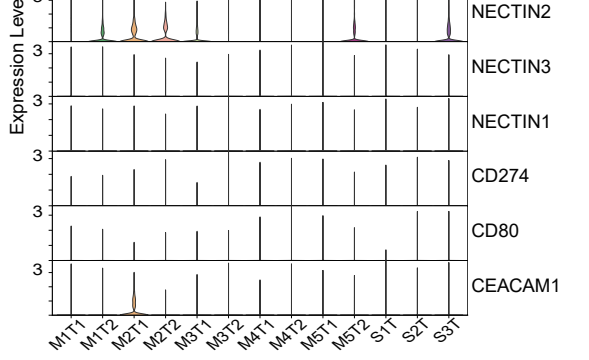

1

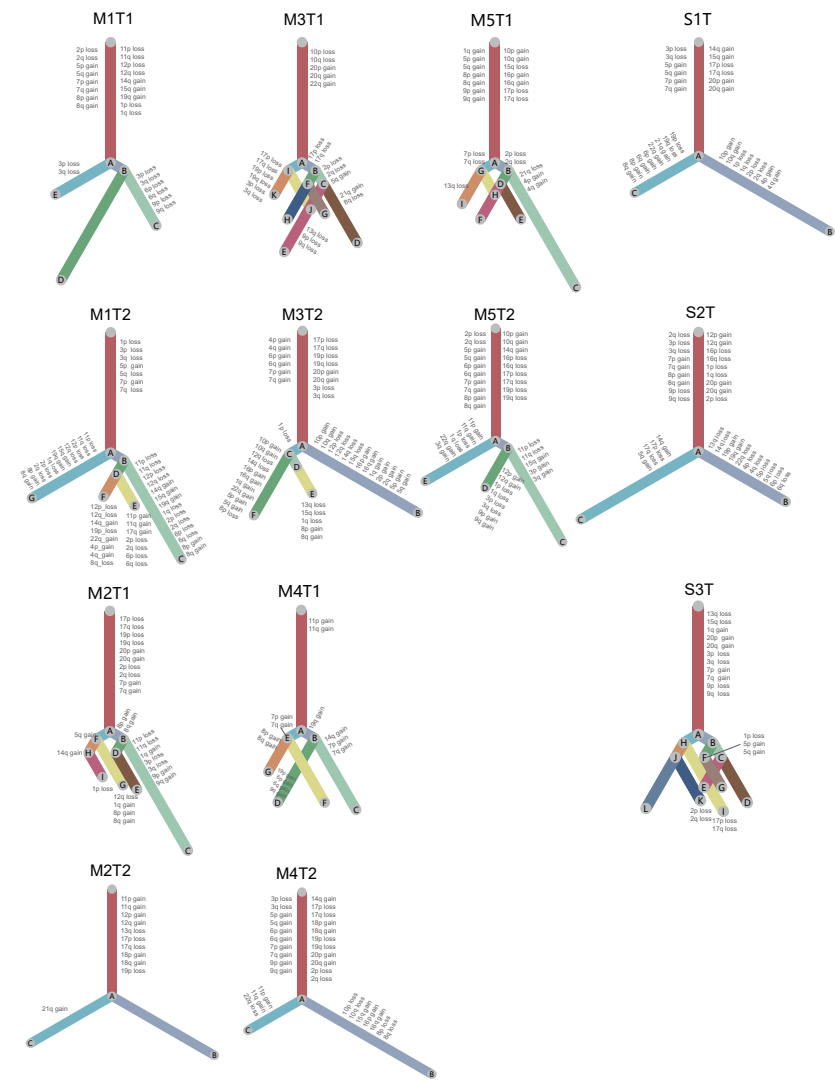

9

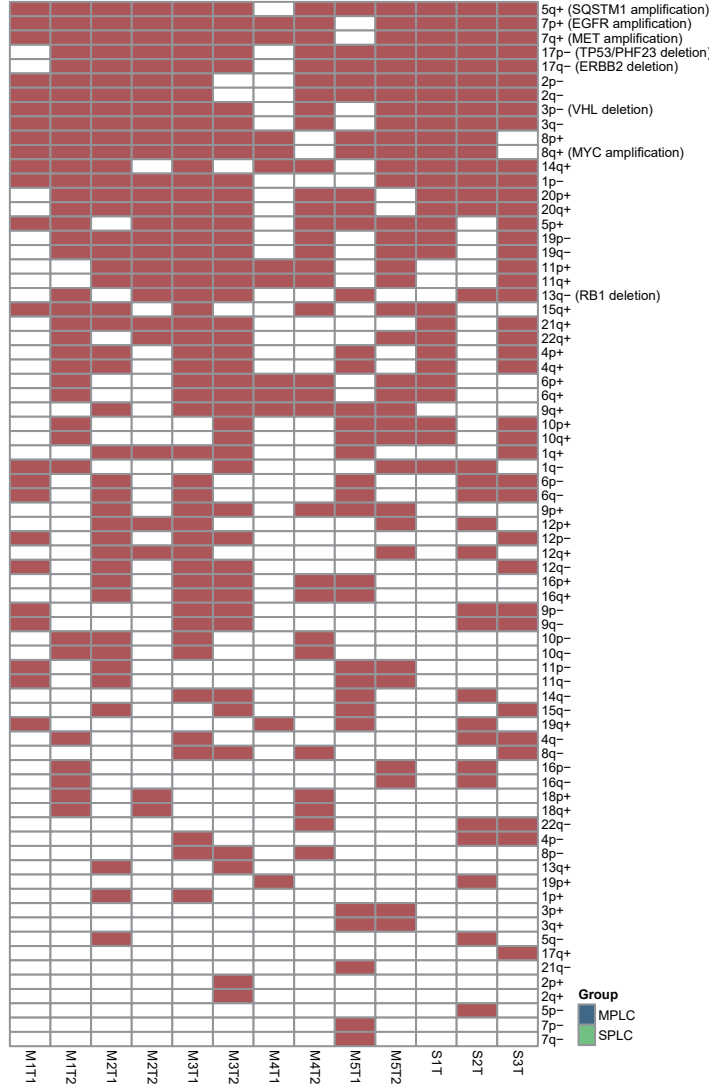

Supplement: Supplementary file 3 — FIGURE S2 Genomic and transcriptomic analysis of epithelial cell lineages in MPLCs and SPLCs. (A) Representative CNV heatmaps obtained from inferCNV analysis grouped by tumour lesion. Each column represents a single cell, and each row represents a different chromosomal region. (B) Violin plots showing distributions of CNV scores among different groups, including epithelial cells from MPLCs, SPLCs and normal tissues. (C) Venn diagram showing the overlap of differentially expressed genes (DEGs) in malignant cells of MPLCs and SPLCs compared to epithelial cells from normal tissues. The top part of the diagram shows the overlap of up‐regulated DEGs, while the bottom part shows the overlap of down‐regulated DEGs. Thirty‐six up‐regulated and 64 down‐regulated DEGs common to both MPLCs and SPLCs. (D) Gene Set Enrichment Analysis (GSEA) analysis showing significant enrichment of genes with higher expression in malignant cells of MPLCs (top) and SPLCs (bottom) compared to epithelial cells from normal tissues. The enriched pathways include hallmarks of epithelial–mesenchymal transition (EMT), p53 pathway. (E) Stacked violin plot showing the expression levels of immune checkpoint molecules across different tumour samples. (F) Clonality trees for each of the 13 tumour lesion. The branches of the trees are scaled according to the percentage of cells present in each subclone with the corresponding CNV event. Each tree represents the clonal architecture and evolution of the tumour lesions. (G) Summary plot of the CNV events from each of the 13 tumour lesions inferred from their scRNA‐seq data. The plot shows the presence or absence of CNV events across different chromosomal regions for each tumour lesion, grouped by MPLC and SPLC. [file CTM2-14-e70091-s003.pdf]

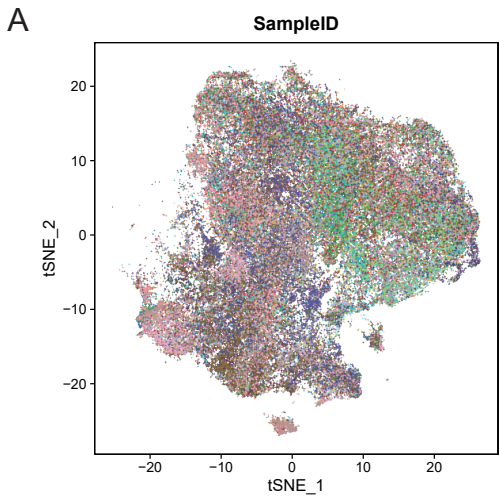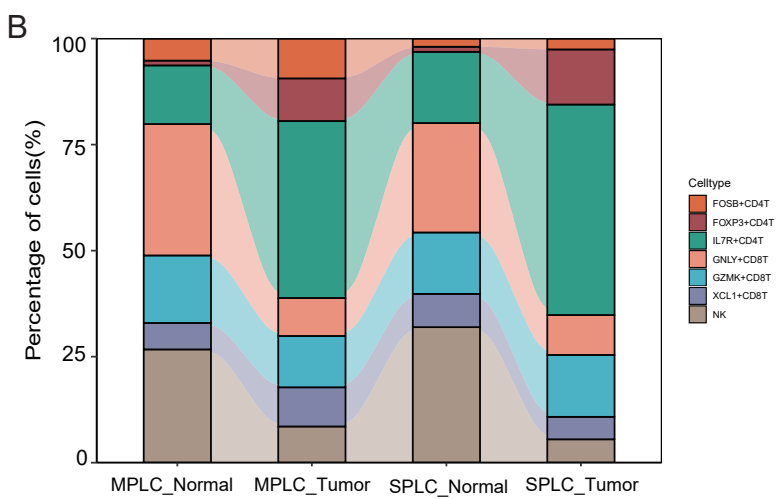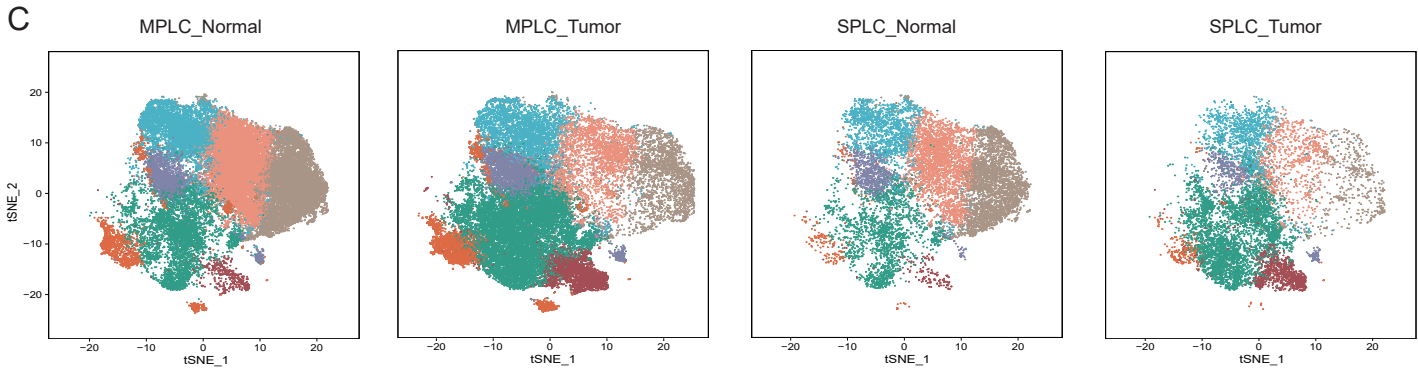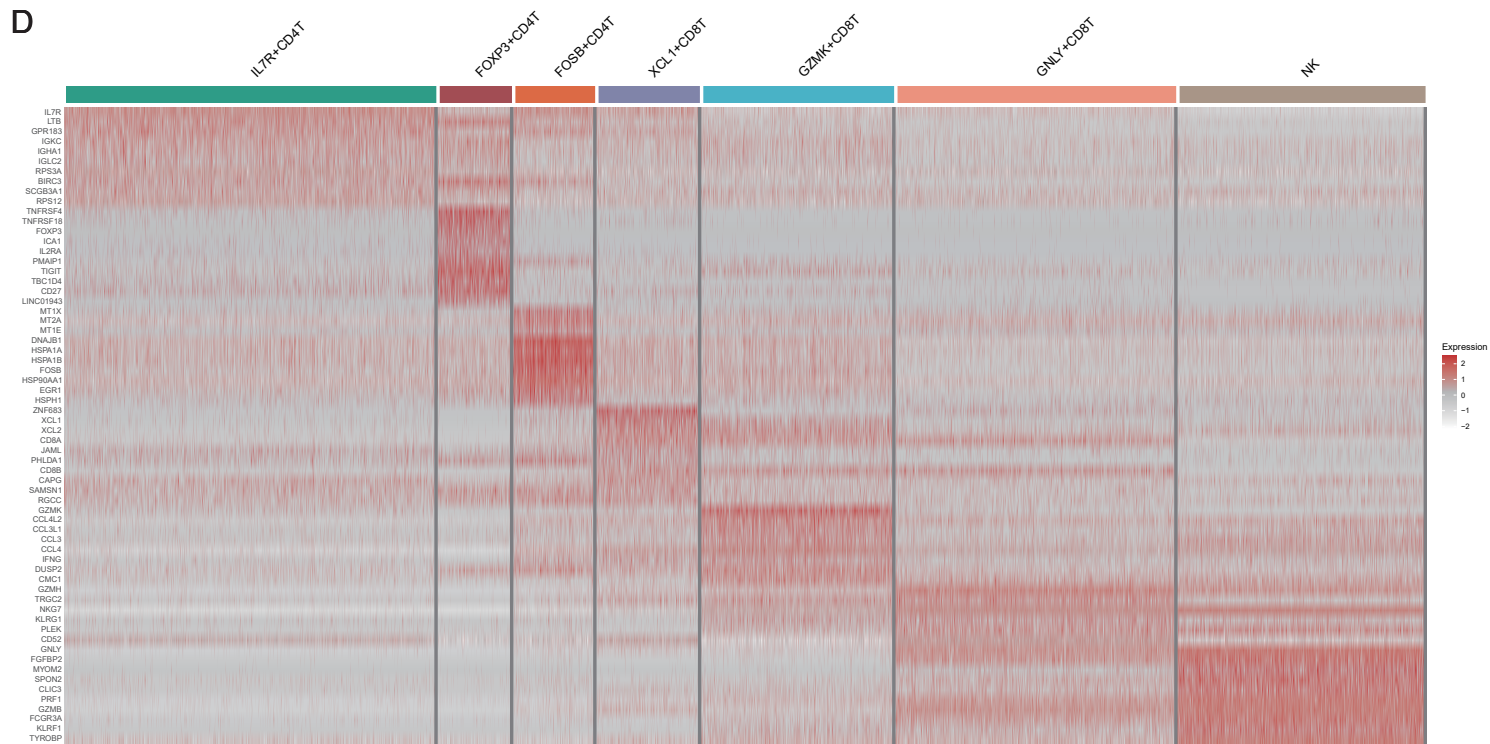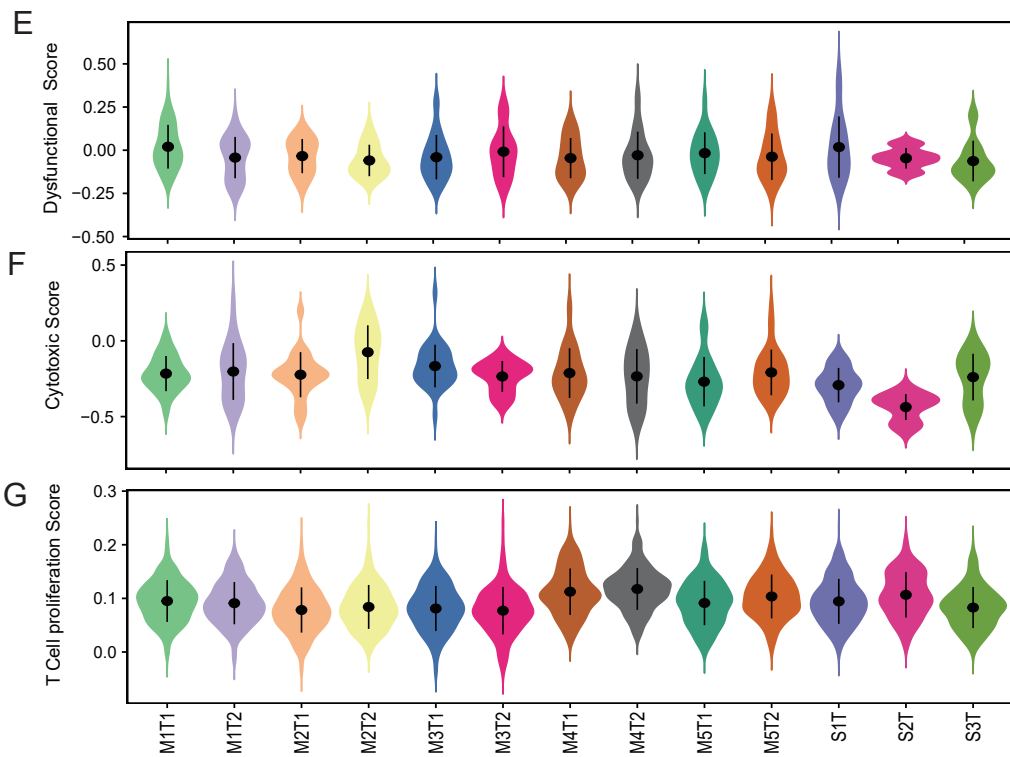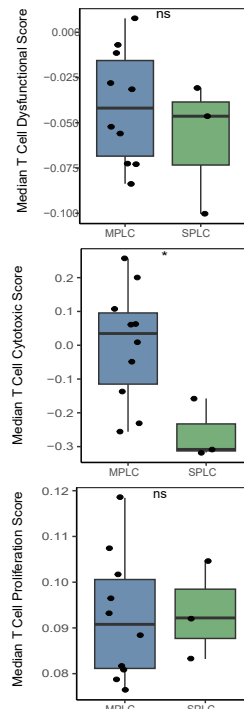

Supplement: Supplementary file 4 — FIGURE S3 T/NK cell subsets, related to Figure 2. (A) t‐SNE plot illustrating the clustering of 90 152 T/NK cells, with each cell colour‐coded by its corresponding sample ID. (B) Bar plot depicting the fractions of T/NK cell types detected in each group, coloured‐coded by major T/NK cell lineages as shown in Figure 3. (C) t‐SNE projections within each group, colour‐coded by T/NK cell lineages. (D) Heatmap showing the top 10 differentially expressed genes (rows) according to the log2FC across T/NK cell types (columns). The heatmap is organised by T/NK cell lineages, with genes colour‐coded to indicate their relative expression levels. (E) Violin plots displaying the dysfunction scores of CD8+ Tex cells across different samples (M1T1–S3T). The right panel shows a box plot comparing the median T cell dysfunction scores between MPLC and SPLC groups, indicating no significant difference (ns). (F) Violin plots showing the cytotoxic scores of CD8+ Teff cells across different samples (M1T1–S3T). The right panel presents a box plot comparing the median T cell cytotoxic scores between MPLC and SPLC groups, demonstrating significantly higher scores in MPLC. (G) T cell proliferation scores: Violin plots depicting the proliferation scores of T cells across different samples (M1T1–S3T). The right panel illustrates a box plot comparing the median T cell proliferation scores between MPLC and SPLC groups, indicating no significant difference (ns). [file CTM2-14-e70091-s005.pdf]

A

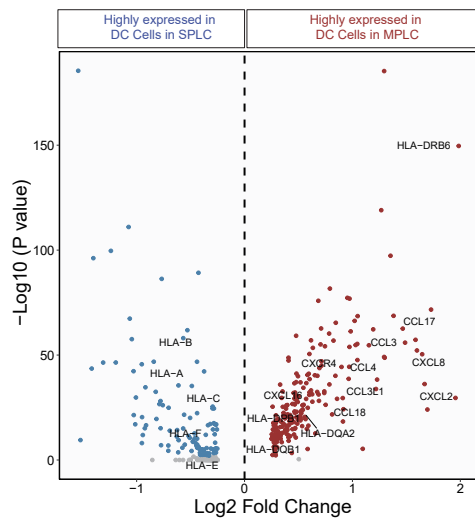

B

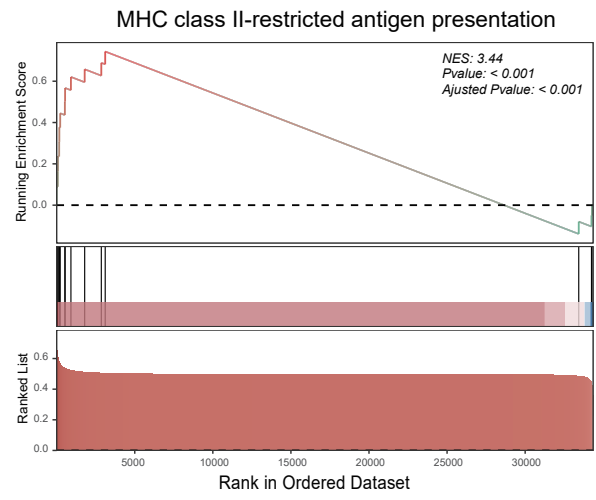

C

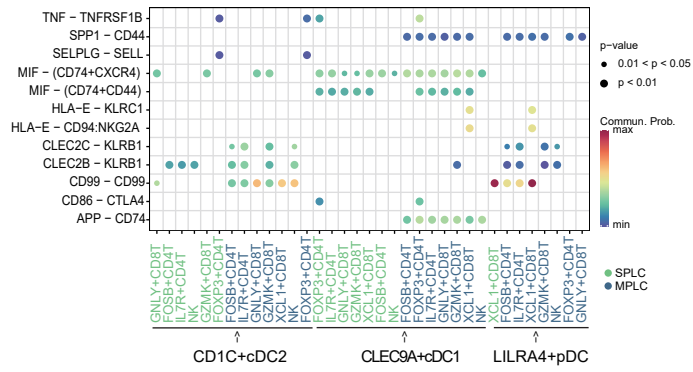

D

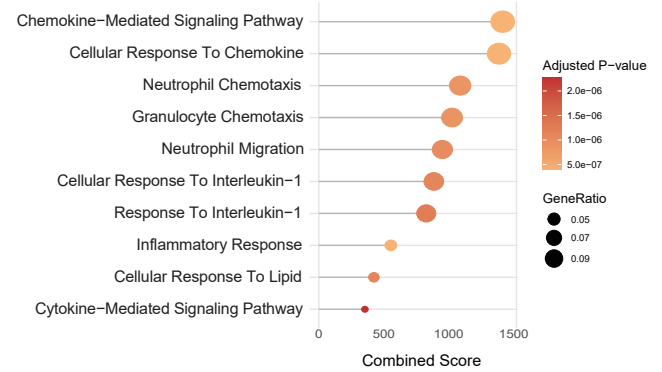

E

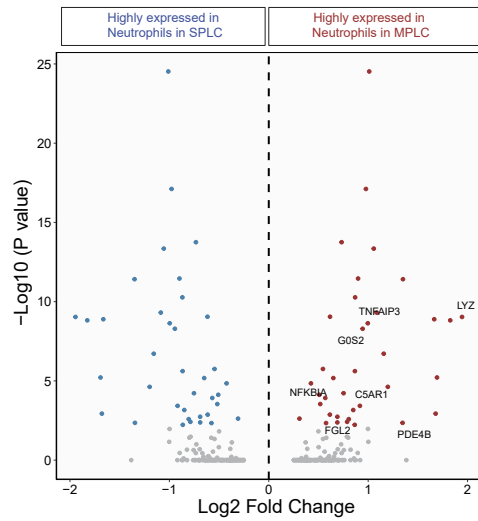

F

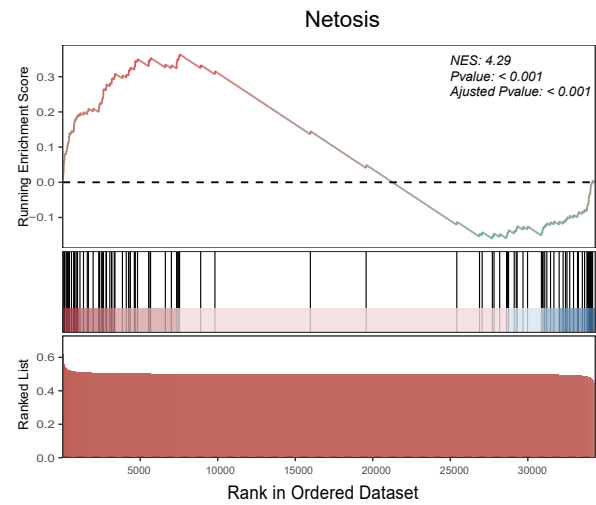

G

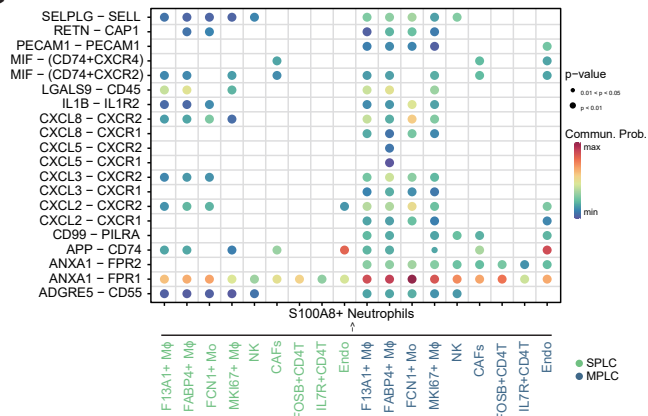

Supplement: Supplementary file 6 — FIGURE S5 Characterisation of dendritic cells (DCs) and neutrophils. (A) Volcano plot showing differentially expressed genes between DCs from MPLC and SPLC. Red dots represent up‐regulated genes in MPLC DCs, and blue dots represent up‐regulated genes in SPLC DCs. (B) Gene Set Enrichment Analysis (GSEA) indicating that genes with higher expression in DCs from MPLCs are significantly enriched in the MHC class II‐restricted antigen presentation pathway. (C) Dot plot illustrating the communication between T/NK cells and DCs in MPLC and SPLC. The size of the dots represents the p value of the interaction, and the colour indicates the communicating probability. (D) The bubble plot displaying the top 10 enriched GO terms in cDC2 cells from MPLC samples. The x‐axis represents the combined score; the y‐axis lists the GO terms. The size of each bubble corresponds to the GeneRatio, and the colour indicates the adjusted p value, with darker shades representing more significant p values. (E) Volcano plot illustrating differentially expressed genes between neutrophils from MPLCs and SPLCs. Genes with significantly higher expression in MPLC neutrophils are shown in red, while those with higher expression in SPLC neutrophils are shown in blue. (F) Gene Set Enrichment Analysis revealed an enrichment of genes with higher expression in neutrophils from MPLCs, compared to those from SPLCs, in the NETosis pathway. (G) Dot plot depicting the communication between neutrophils and other cell types in both MPLC and SPLC tumours. The size of the dots represents the statistical significance (p value), with larger dots indicating stronger significance. The colour intensity represents the communication probability. [file CTM2-14-e70091-s008.pdf]

A

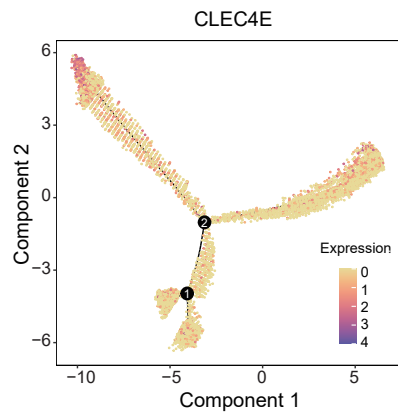

B

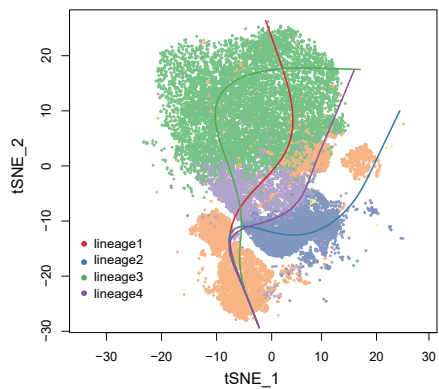

C

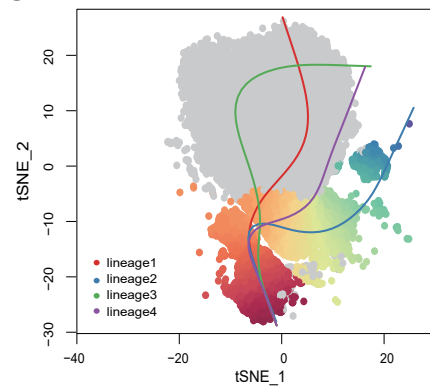

D

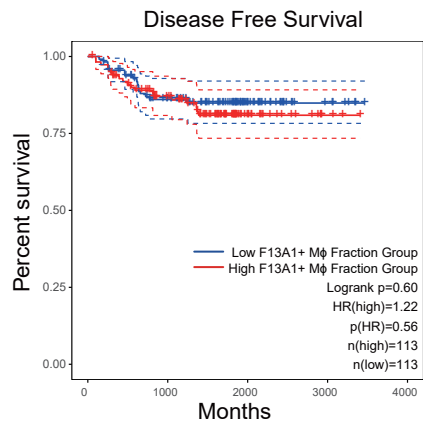

Supplement: Supplementary file 7 — FIGURE S6 Trajectory and prognostic analysis of F13A1+ Mϕ. (A) Expression of the early macrophage‐specific gene CLEC4E along the Monocle2 differentiation trajectory. (B) Trajectory analysis of Mo/Mϕ populations using Slingshot, showing four predicted lineages, each represented by a distinct colour. The red trajectory corresponds to Lineage 1, which represents the transition from FCN1+ Mo to EMP1+ Mϕ. Lineage 2 is shown in blue, indicating the path from FCN1+ Mo to F13A1+ Mϕ. The green trajectory represents Lineage 3, illustrating the transition from FCN1+ Mo to FABP4+ Mϕ while Lineage 4, displayed in purple, depicts the path from FCN1+ Mo to LYVE1+ Mϕ. These trajectories capture the potential differentiation paths inferred by Slingshot. (C) Visualisation of cells along Lineage 2 (FCN1+ Mo to F13A1+ Mϕ), with cell colours representing pseudotime progression. Cells are coloured from red (early pseudotime) to blue (late pseudotime), reflecting their position along the pseudotime axis. (D) Kaplan–Meier survival curves showing the disease‐free Survival (DFS) of lung cancer patients in the GSE31210 dataset, stratified by the F13A1+ Mϕ fraction. Patients were grouped based on the median cell fraction, comparing high and low expression levels. The curves highlight the impact of F13A1+ macrophage fraction on disease progression and patient outcomes. [file CTM2-14-e70091-s006.pdf]

A

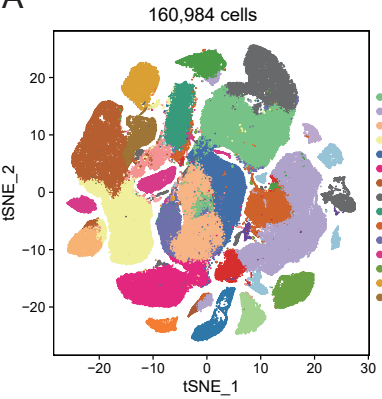

B

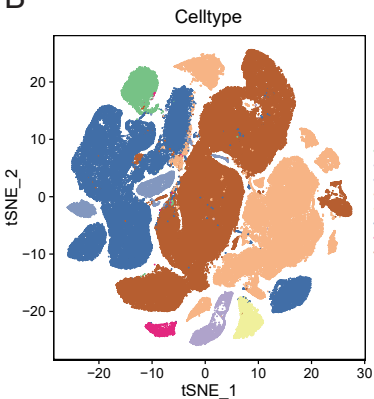

C

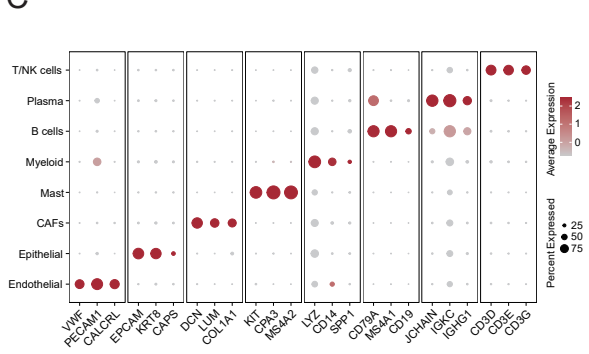

D

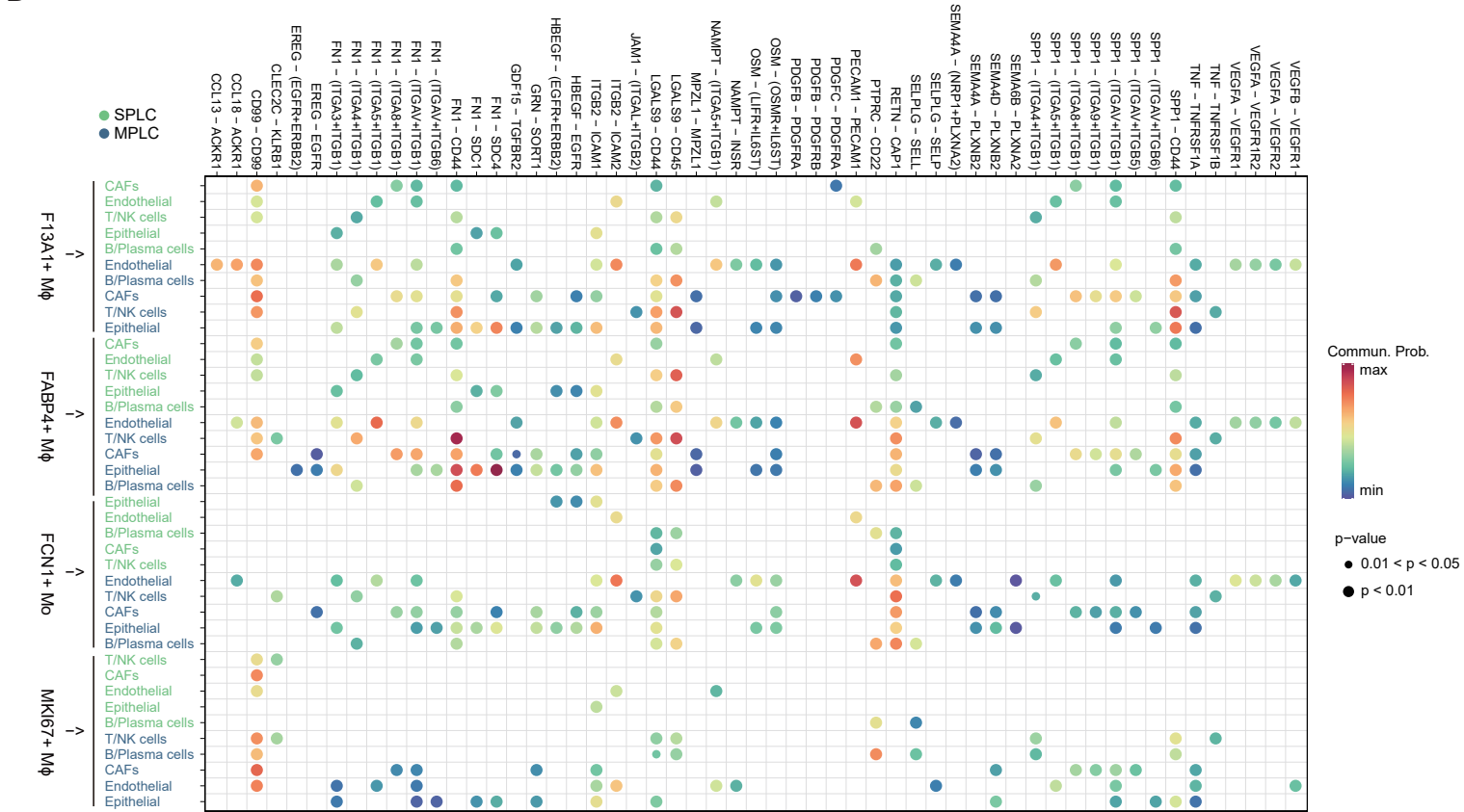

Supplement: Supplementary file 8 — FIGURE S7 Validation of findings in external datasets. (A) t‐SNE plot showing the clustering of 160 984 cells from external datasets (GSE200972, E‐MTAB‐6149 and E‐MTAB‐6653) of MPLC and SPLC. Each colour represents a different cluster. (B) t‐SNE projection of cell types from the external data, annotated with the inferred cell types. (C) Dot plot displaying selected cell type‐specific markers across all cell types. The dot size represents the fraction of cells expressing a particular marker, and the colour intensity indicates the average expression level. (D) Dot plot illustrating the communication between Mo/Mϕs and other cell types in MPLC and SPLC, based on CellChat validation in external datasets. The size of the dots represents the p value of the interaction, and the colour indicates the communicating probability. [file CTM2-14-e70091-s007.pdf]
