# Supplementary material for: Single‐cell dissection reveals immunosuppressive F13A1+ macrophage as a hallmark for multiple primary lung cancers
Source: Clin Transl Med. 2024 Nov 27;14(12):e70091. doi: 10.1002/ctm2.70091 (PMC11600049; doi:10.1002/ctm2.70091)
Supplement: Supplementary file 5 — FIGURE S4 Myeloid cell subsets, related to Figure 3. (A) t‐SNE plot illustrating the clustering of 118 556 myeloid cells, with each cell colour‐coded by its corresponding sample ID. (B) Bar plot depicting the fractions of myeloid cell types detected in each group, coloured‐coded by major cell lineages as in Figure 4. (C) t‐SNE projection within each group, colour‐coded by myeloid cell lineages. (D) Heatmap displaying the top 10 differentially expressed genes (rows) according to the log2FC across myeloid cell types (columns). The heatmap is organised by major cell lineages, with genes colour‐coded to indicate their relative expression levels. [file CTM2-14-e70091-s004.pdf]

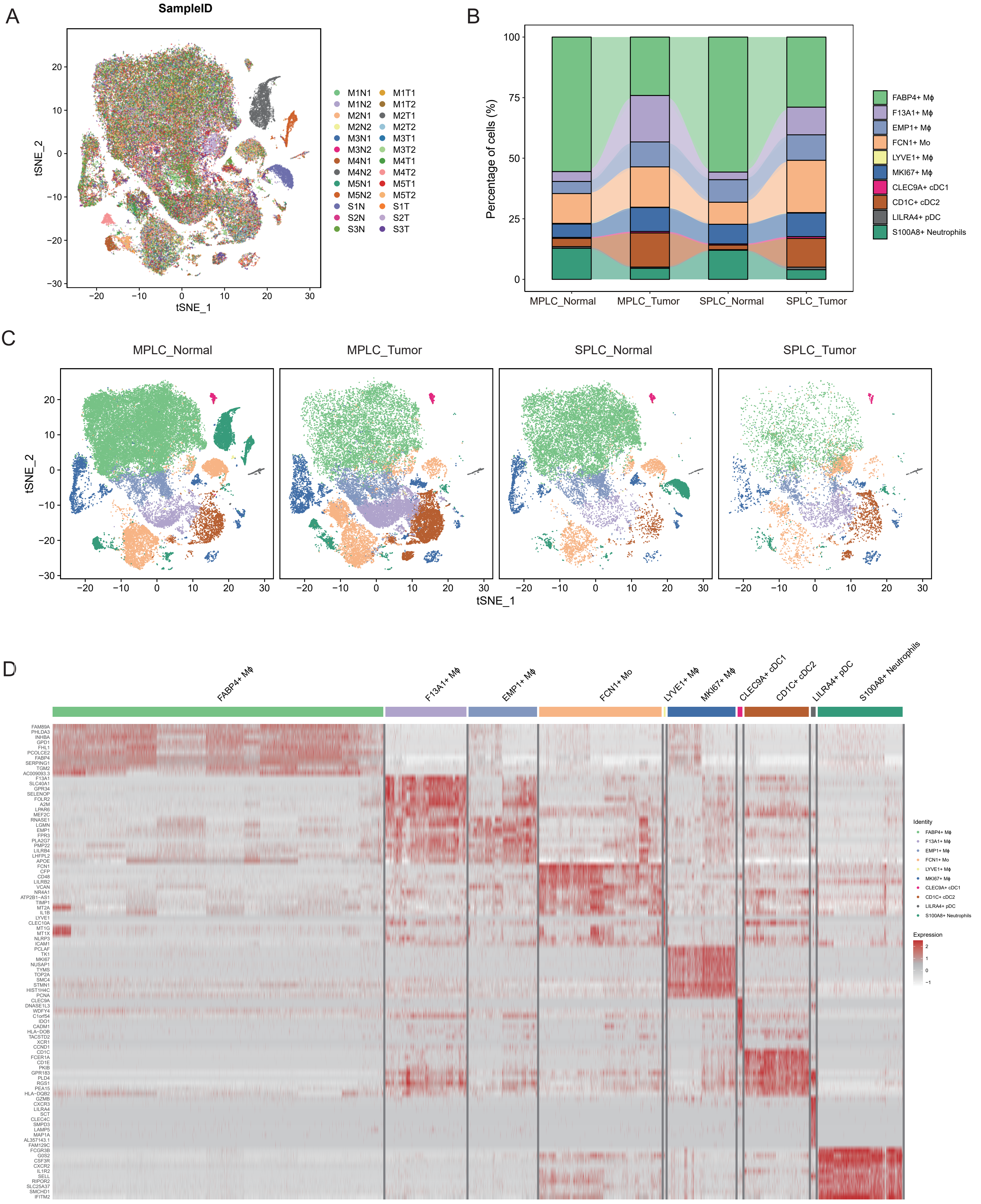
A
SampleID
tSNE_2
tSNE_1
M1N1
M1N2
M2N1
M2N2
M3N1
M3N2
M4N1
M4N2
M5N1
M5N2
S1N
S2N
S3N
M1T1
M1T2
M2T1
M2T2
M3T1
M3T2
M4T1
M4T2
M5T1
M5T2
S1T
S2T
S3T
B
Percentage of cells (%)
MPLC_Normal
MPLC_Tumor
SPLC_Normal
SPLC_Tumor
FABP4+ Mϕ
F13A1+ Mϕ
EMP1+ Mϕ
FCN1+ Mo
LYVE1+ Mϕ
MKI67+ Mϕ
CLEC9A+ cDC1
CD1C+ cDC2
LILRA4+ pDC
S100A8+ Neutrophils
C
MPLC_Normal
MPLC_Tumor
SPLC_Normal
SPLC_Tumor
tSNE_2
tSNE_1
D
FABP4+ Mϕ
F13A1+ Mϕ
EMP1+ Mϕ
FCN1+ Mo
LYVE1+ Mϕ
MKI67+ Mϕ
CLEC9A+ cDC1
CD1C+ cDC2
LILRA4+ pDC
S100A8+ Neutrophils
FAM89A
PHLDA3
INHBA
GPD1
FHL1
PCOLCE2
FABP4
SERPING1
TGM2
AC009093.3
F13A1
SLC40A1
GPR34
SELENOP
FOLR2
A2M
LPAR6
MEF2C
RNASE1
LGMN
EMP1
FPR3
PLA2G7
PMP22
LILRB4
LHFPL2
APOE
FCN1
CFP
CD48
LILRB2
VCAN
NR4A1
ATP2B1-AS1
TIMP1
MT2A
IL1B
LYVE1
CLEC10A
MT1G
MT1X
NLRP3
ICAM1
PCLAF
TK1
MKI67
NUSAP1
TYMS
TOP2A
SMC4
STMN1
HIST1H4C
PCNA
CLEC9A
DNASE1L3
WDFY4
C1orf54
IDO1
CADM1
HLA-DOB
TACSTD2
XCR1
CCND1
CD1C
FCER1A
CD1E
PKIB
GPR183
PLD4
RGS1
PEA15
HLA-DQB2
GZMB
CXCR3
LILRA4
SCT
CLEC4C
SMPD3
LAMP5
MAP1A
AL357143.1
FAM129C
FCGR3B
G0S2
CSF3R
CXCR2
IL1R2
SELL
RIPOR2
SLC25A37
SMCHD1
IFITM2
Identity
Expression
